# Theoretical Description of Infrared Near-Field Spectroscopy of In- and Out-of-Plane Molecular Vibrations in Thin Layers

**DOI:** 10.1021/acsphotonics.5c00798

**Published:** 2025-06-27

**Authors:** Isabel Pascual Robledo, Carlos Maciel-Escudero, Martin Schnell, Lars Mester, Javier Aizpurua, Rainer Hillenbrand

**Affiliations:** † Material Physics Center, CSIC-UPV/EHU, Paseo de Manuel Lardizabal 5, Donostia-San Sebastian 20018, Spain; ‡ 138823 CIC nanoGUNE BRTA, Tolosa Hiribidea, Donostia-San Sebastian 20018, Spain; § 226245 Donostia International Physics Center, Paseo de Manuel Lardizabal 3, Donostia-San Sebastian 20018, Spain; ∥ IKERBASQUE, Basque Foundation of Science, Bilbao 48011, Spain; ⊥ 609501 Attocube Systems GmbH, Eglfinger Weg 2, 85540 Haar, Germany; # Dept. of Electricity and Electronics, University of the Basque Country (UPV/EHU), Leioa 48940, Spain

**Keywords:** infrared nanospectroscopy, s-SNOM, nano-FTIR spectroscopy, thin organic layers, in- and out-of-plane molecular vibrations, spectral peak shifts

## Abstract

Infrared nanospectroscopy, based on scattering-type scanning near-field optical microscopy (s-SNOM), allows for chemical nanoidentification of organic composite layers by local probing of their molecular vibrations. However, the conditions for probing in-plane and out-of-plane molecular vibrations by this technique remain largely unexplored. Here, we perform a systematic theoretical study of the local infrared near-field response of isotropic and anisotropic thin layers using electrostatic numerical calculations, complemented by analytical electrodynamic point-dipole model calculations. Specifically, we study uniaxial thin layers exhibiting molecular vibrations with different orientations on highly and weakly reflecting substrates. We find that both in-plane and out-of-plane molecular vibrations can be probed, with the sensitivity to in-plane vibrations being reduced in samples where the near fields are vertically oriented, such as in thin layers on highly reflecting substrates. We finally show that fast calculations of near-field spectra of uniaxial thin layers can be done with a perturbative finite-dipole model, achieving reasonable quantitative accuracy compared to electrostatic numerical results.

## Graphical abstract

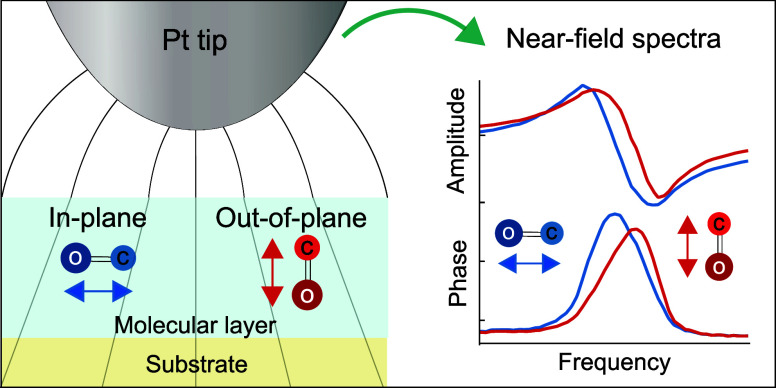

## Introduction

Infrared (IR) spectroscopy is a powerful and widely used technique for characterizing the chemical composition and structural properties of materials. It provides valuable insights into molecular vibrations and electronic excitations, making it an essential tool across various scientific disciplines. However, conventional far-field IR spectroscopy is fundamentally limited by optical diffraction, which prevents spatial resolution on the nanoscale and restricts its applicability to heterogeneous or nanostructured materials.

A technique that circumvents the diffraction limit and enables nanoscale-resolved IR spectroscopy is scattering-type scanning near-field optical microscopy (s-SNOM).^1–4^ s-SNOM is based on atomic force microscopy (AFM), where a sharp metallic AFM tip is illuminated and acts as an optical antenna.^5,6^ The tip focuses the incident p-polarized light into a highly confined near field at the tip apex, referred to as the nanofocus. Due to near-field interaction between tip and sample, the tip-scattered light encodes the local optical properties of the sample. By scanning the sample while performing amplitude- and phase-resolved detection^7^ of the elastically scattered light, s-SNOM provides near-field optical images with spatial resolution down to 10 nm, far beyond the diffraction limit. Background scattering is suppressed by vertically oscillating the AFM tip and demodulating the detector signal at higher harmonics of the tip oscillation frequency. Beyond imaging, s-SNOM can be extended to spectroscopic measurements by either recording near-field images at different wavelengths or by employing broadband laser sources combined with, for example, Fourier transform spectroscopy (nano-FTIR spectroscopy) to obtain local IR spectra.^2,3,8–14^

When probing molecular vibrations or other weak oscillators, near-field amplitude spectra approximately correspond to far-field reflection spectra, whereas near-field phase spectra can be interpreted as absorption spectra.^3,15^ However, this is not always accurate, as spectral peak shifts between near-field and far-field spectra commonly occur.^16,17^ These shifts complicate the interpretation of the spectra, and modeling is required for a quantitative analysis of the exact peak positions and peak shapes.^15,17–30^ The origin and evolution of spectral peak shifts in near-field spectra, influenced by parameters like sample thickness or substrate properties, remains largely unexplored. Further, the role of the orientation of molecular vibrations is under discussion, specifically whether in-plane-oriented molecular vibrations can be detected and under which conditions.^12,31–41^ A systematic investigation of these issues is therefore crucial for advancing the understanding and quantitative interpretation of nanoscale IR spectroscopy of molecular vibrations.

Here, we present a comprehensive theoretical study to elucidate the capability of infrared nanospectroscopy based on s-SNOM to probe in- and out-of-plane-oriented molecular vibrations in organic samples. To this end, we perform electrostatic numerical calculations of infrared near-field spectra of such samples, incorporating experimental details such as the elongated tip shape, vertical tip oscillation and signal demodulation procedure. The samples under study are composed of anisotropic layers of varying thicknesses over both weakly and highly reflecting substrates. These anisotropic layers are described by uniaxial dielectric tensors, representing three different configurations: (i) isotropic, (ii) in-plane- and (iii) out-of-plane-oriented molecular vibrations. We find that the elongated tip is sensitive to both in- and out-of-plane molecular vibrations. In-plane vibrations can be probed both in thick layers and in thin layers on weakly reflecting substrates. However, the sensitivity to in-plane vibrations is strongly reduced when the anisotropic layer is placed on highly reflecting substrates, as the near fields induced within the layer are oriented vertically with respect to its surface. We further observe spectral shifts of molecular features in the calculated near-field spectra that can be understood by analyzing the Fresnel reflection coefficients of the multilayer. Our numerical results are qualitatively confirmed by results obtained with an analytical electrodynamic point-dipole model (PDM) extended to account for uniaxial thin layers. Additionally, our results can be reproduced by adapting a perturbative finite-dipole model (FDM), which provides quantitative accuracy in addition to being considerably faster than the numerical calculations.

## Results and Discussion

We study the infrared near-field spectral response of anisotropic thin layers with molecular vibrations using numerical electrostatic calculations implemented in the AC/DC Module of COMSOL Multiphysics software.^42^ Electrostatic calculations are generally faster than the standard electrodynamic calculations implemented in the COMSOL Wave Optics Module.^43^ Specifically, the electrostatic calculations performed in this study are 5 to 10 times faster than their corresponding electrodynamic versions, while providing quantitatively similar results. We demonstrate the quantitative agreement between the two methods in Supporting Information (SI) section 1. Note that far-field reflection at the sample surface is not considered in any of the calculations, as it cannot be directly implemented in the electrostatic model. Our electrostatic model (illustrated in Figure 1a) is an adaptation from the model described in ref 23. We consider a semiellipsoidal platinum tip (depicted in gray), positioned at a tip–sample distance *h* above a layer of thickness *d* and characterized by a dielectric tensor *ε⃡*(ν) on a substrate (depicted in blue and yellow, respectively). The incident illumination is defined as a constant vertical electrostatic field ${\mathrm{E⃗}}_{\mathrm{inc}}=E_{\mathrm{inc}}ẑ$ , with magnitude *E*_inc_ = 1 V/m (see Methods).

**Figure 1:**
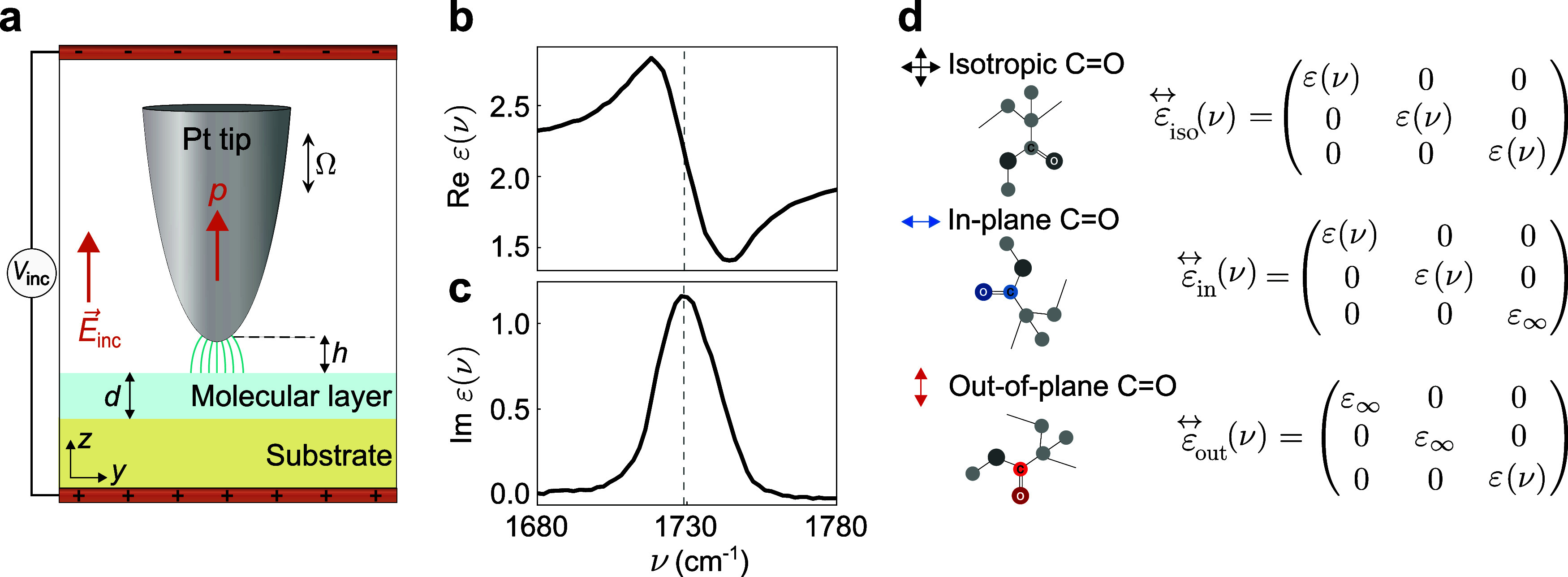
Geometrical configuration and dielectric response for electrostatic numerical calculations of infrared near-field spectroscopy of anisotropic samples. (a) Schematic representation of the simulation configuration. The platinum tip (gray) is modeled as a semiellipsoid, positioned at a tip–sample distance *h* above a molecular layer (blue) of thickness *d*, which sits on a substrate (yellow). A constant vertical electric field (orange arrow) *E⃗*_inc_ = *E*_inc_ẑ (*E*_inc_ = 1 V/m), set by an electrostatic potential *V*_inc_, polarizes the tip. The resulting near fields at the tip apex interact with the sample and modify the polarization of the tip, which leads to a total dipole moment *p* (orange arrow). (b) Real and (c) imaginary parts of the dielectric function of PMMA, ε­(ν). The curves showcase the PMMA carbonyl (C=O) bond stretching vibration around ν = 1730 cm^–1^ (gray-dashed line). (d) Molecular diagrams and dielectric tensors for the different anisotropic samples. From top to bottom: isotropic sample, ε⃡_iso_(ν) (black crossed arrows); sample with exclusively in-plane vibrations, ε⃡_in_(ν) (blue horizontal arrow); and sample with exclusively out-of-plane vibrations, ε⃡_out_(ν) (red vertical arrow). The molecular representations help to visualize the molecular vibration orientation. In all cases ε_∞_ = 2.145 represents the high-frequency dielectric value of PMMA.

We study three exemplary types of layers with dielectric tensors that include the PMMA C=O vibrational stretching mode, characterized by the dielectric function ε­(ν) from ref 44 (shown in Figure 1b,c), where ν is the frequency in cm^–1^. The dielectric tensors for these layers are constructed by assigning ε­(ν) to their different diagonal components, as illustrated in Figure 1d. In the isotropic case, the dielectric function ε­(ν) is present in all diagonal components of the tensor ε⃡_iso_(ν), such that ε_*xx*_ = ε_*yy*_ = ε_*zz*_ = ε­(ν). For the second case, ε­(ν) is present only in the two in-plane components of the tensor ε⃡_in_ (ν), as ε_*xx*_ = ε_*yy*_ = ε­(ν). Finally, for the third case, ε­(ν) is exclusively present in the out-of-plane component of the tensor ε⃡_out_(ν), that is, *ε*_*zz*_ = ε­(ν). The remaining diagonal components of ε⃡_in_(ν) and ε⃡_out_(ν) only account for the high-frequency dielectric value of the PMMA C=O vibration, that is ε_∞_ = 2.145. Within this approach, the tensor components containing ε­(ν) represent the presence of the vibrational resonance along the corresponding spatial direction. Since the same ε­(ν) (with identical oscillator strength) is used, we can directly compare the infrared response of the three different layers. The overall infrared response of each layer depends on the number of tensor components that include ε­(ν), making the isotropic case the one with the strongest infrared response.

Our calculations of amplitude and phase of the near-field spectra incorporate higher harmonic demodulation used in s-SNOM experiments, where the tip oscillates vertically with frequency Ω and the signal is detected at higher harmonics *n*Ω (with *n* ≥ 2). To that end, we first calculate the tip-scattered field *E*_sca_(*h*,ν) for various tip–sample distances *h* and frequencies ν. This scattered field is proportional to the dipole moment induced at the tip, *p*(*h*,ν), determined by the first moment of the surface charge distribution along the tip surface (see Methods). As the tip undergoes a vertical sinusoidal motion *h*(*t*) = *h*_0_ + *A*[1 – cos­(Ω*t*)] with amplitude *A* and minimum tip–sample distance *h*_0_, we express the tip-scattered field as a periodic function of time, *E*_sca_(*h*(*t*),ν). Next, we calculate the *n*-th Fourier coefficient of the tip-scattered field *E*_sca,*n*_(ν) and normalize it to that obtained on a reference material (gold in our case), *E*_ref,*n*_(ν). This yields the normalized near-field amplitude and phase spectra *s_n_* (ν) = |*E*_sca,*n*_(ν)|/|*E*_ref,*n*_(ν)| and φ_*n*_(ν) = Arg­[*E*_sca,*n*_(ν)] – Arg­[*E*_ref,*n*_(ν)], respectively.

Figure 2 compares the calculated near-field spectra for thin molecular layers on highly (Au) and weakly (CaF_2_) reflecting substrates. Figure 2a,b show the normalized amplitude *s*_2_(ν) and phase φ_2_(ν) spectra for both the isotropic layer (black) and the layer with exclusively out-of-plane-oriented molecular vibrations (red) on a Au substrate. The amplitude spectra exhibit a dispersive line shape, and the phase spectra exhibit a resonance peak near the molecular vibrational frequency ν = 1730 cm^–1^, respectively. This behavior closely resembles the real and imaginary part of the dielectric function ε­(ν) associated with the vibration,^26^ as also experimentally observed in refs 15–17 . Conversely, for the layer on Au with exclusively in-plane-oriented molecular vibrations (blue curves in Figure 2a,b), the molecular spectral features nearly vanish, as experimentally indicated in ref 31. To understand this finding, Figure 2c,d show the absolute value of the in- and out-of-plane near-field components around the tip that is located above a thin isotropic layer on Au, |*E*_*y*_| and |*E*_*z*_|, respectively. We observe that the out-of-plane near-field component is significantly stronger inside the molecular layer, while the in-plane component nearly vanishes close to the Au substrate. This results from the boundary conditions imposed by the metallic substrate, which enforce the electric field lines in the layer to orient normal to the Au surface.^45^ Consequently, in-plane-oriented molecular vibrations are barely excited by the electric near field. A similar behavior is observed with Si substrates (SI section 2), which are commonly used in s-SNOM studies. Due to the large permittivity of Si (ε_Si_ ≈ 11.7),^46^ the electric field lines are predominantly oriented nearly perpendicular to the sample surface, similar to the case of Au substrates.

**Figure 2:**
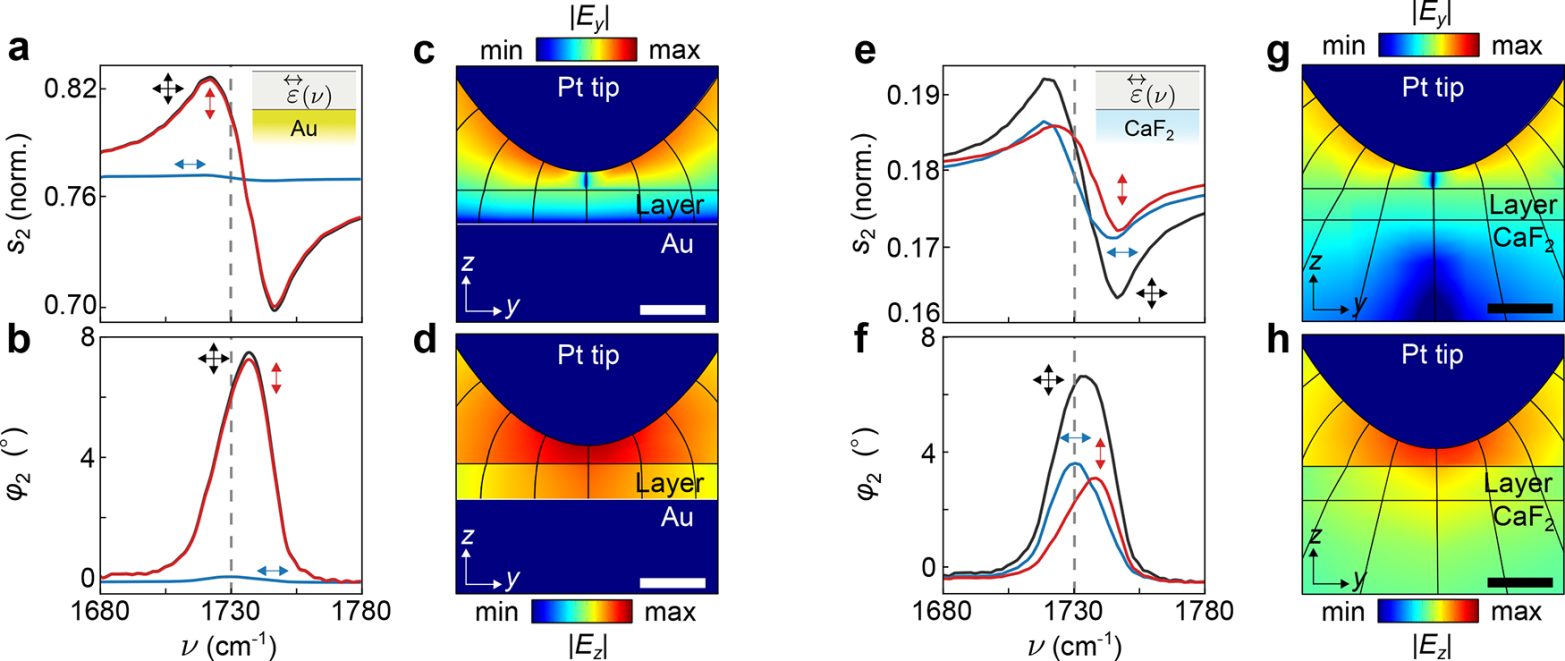
Infrared near-field spectra of anisotropic thin layers on highly and weakly reflecting substrates. (a) Calculated near-field amplitude *s*_2_(ν) and (b) phase φ_2_(ν) spectra of a 5 nm-thin layer with ε⃡_iso_(ν) (black), ε⃡_in_(ν) (blue) and ε⃡_out_(ν) (red) dielectric tensors on an Au substrate. The vertical dashed line indicates the molecular vibration frequency around ν = 1730 cm^–1^. Black, blue, and red arrows represent the orientation of the vibration. Inset in panel (a) show the molecular layer (in gray) on top of the Au substrate (in yellow). (c) Color plot of the amplitude of the in-plane near-field component |*E*_*y*_|, and (d) of the out-of-plane near-field component |*E*_*z*_|. The field plots are calculated around the Pt tip apex above a 10 nm-thin isotropic layer at ν = 1730 cm^–1^ on an Au substrate. Black lines in (c, d) represent the electric field lines. (e, f) Same as in (a, b), but for 5 nm-thin anisotropic layers on a CaF_2_ substrate. Inset in panel (e) shows the molecular layer (in gray) on top of the CaF_2_ substrate (in blue). (g, h) Same as in (c, d), but for a 10 nm-thin isotropic layer at ν = 1730 cm^–1^ on a CaF_2_ substrate. Scale bar is 20 nm. Color bar range is from min = 0 V/m to max = 50 V/m.

For comparison, Figure 2e,f shows *s*_2_(ν) and φ_2_(ν) for thin layers on a CaF_2_ substrate. We observe that the spectral features corresponding to the molecular vibration appear not only for the layers with isotropic (black) and out-of-plane-oriented (red) molecular vibrations, but also for the layer with in-plane-oriented (blue) molecular vibrations, as experimentally observed in ref 32. Interestingly, the spectral features for the anisotropic layers are significantly reduced and exhibit small spectral shifts compared to the isotropic layers. These findings will be discussed below. The appearance of the in-plane vibrations in the near-field spectra can be understood by examining |*E*_*y*_| in Figure 2g and |*E*_*z*_| in Figure 2h. We find a largely increased in-plane near-field component compared to Figure 2c,d, which can also be appreciated by the electric field lines (black lines) being tilted relative to the sample surface. The relatively strong in-plane near-field component allows for the excitation and detection of in-plane molecular vibrations on weakly reflecting substrates.

Summarizing the results of Figure 2, we find that the near-field spectral features of thin layers with in-plane molecular vibrations are strongly suppressed when the layer is placed on a highly reflective substrate. This suppression can be understood by the electric field lines in the layer (illustrated in Figure 2c,d for isotropic layers), which are predominantly vertical and thus almost perpendicular to the in-plane-oriented molecular vibrations. In SI section 3, we show that a similar vertical alignment of the field lines also occurs for anisotropic layers, revealing that the orientation of the molecular vibrations does not influence the direction of the electric field lines.

To study the influence of the layer thickness on the spectral features, we compare in Figure 3 near-field spectra of layers with in- and out-of-plane vibrations with thicknesses *d* = 5, 25 and 300 nm. For the layers on CaF_2_ (Figure 3e–h), we observe that the spectral features in both amplitude and phase spectra become more pronounced with increasing thickness *d*. This behavior can be attributed to the presence of more absorbing molecular material in the volume probed by the tip’s near field, analogous to the behavior observed for isotropic layers both theoretically^26^ and experimentally.^16,17,47^ Further, we find that the spectral features of the layers shift slightly toward lower frequencies for out-of-plane vibrations, whereas for in-plane vibrations, they exhibit a pronounced shift toward higher frequencies (compare peak positions in Figure 3f,h). The mechanism behind these shifts is discussed below.

**Figure 3:**
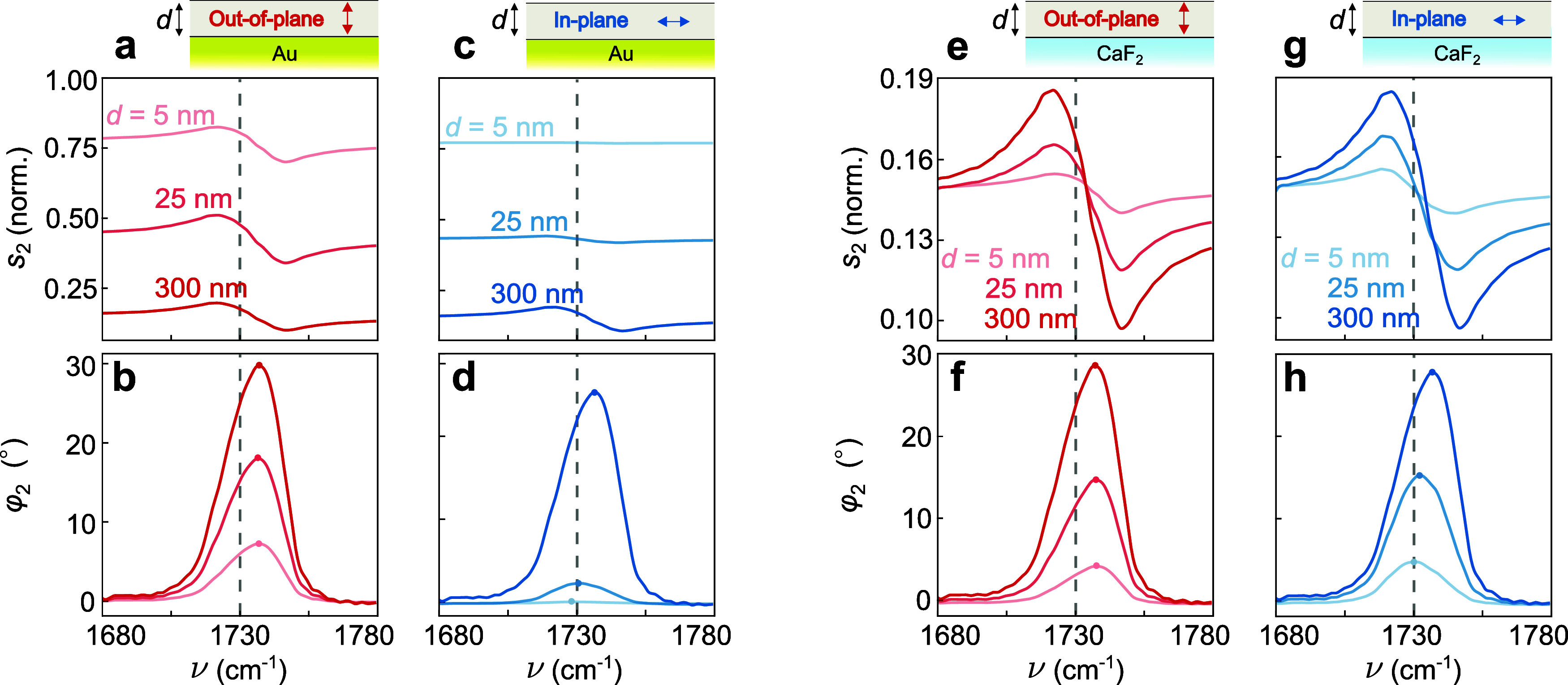
Calculated near-field spectra of anisotropic layers with varying thickness *d*. (a) Calculated near-field amplitude *s*_2_(ν) and (b) phase φ_2_(ν) spectra for layers with ε⃡_out_(ν) and thickness *d* = 5, 25, 300 nm over an Au substrate. The vertical dashed line indicates the molecular vibration frequency around ν = 1730 cm^–1^. (c, d) Same as in (a, b), but for layers with ε⃡_in_(ν) on Au; (e, f) for layers with out-of-plane vibrations on CaF_2_ substrate; (g, h) for layers with in-plane vibrations on CaF_2_ substrate. Sketches above (a, c, e, and g) clarify the bond orientation and the substrate configuration.

For layers on Au (Figure 3a–d), we observe that the spectral features become stronger with increasing layer thickness *d*. Notably, for thin layers with in-plane-oriented molecular vibrations (*d* = 5 nm), the spectral features are negligible, whereas they become comparable to those of layers with out-of-plane-oriented molecular vibrations when the layer thickness reaches 300 nm. This behavior can be attributed to the finite penetration depth of the tip’s near fields—less than 300 nm, as experimentally observed in refs 17 and 48 —preventing near-field interaction between tip and Au substrate. Consequently, the field lines within the molecular layer tilt, similar to their behavior when the layer is placed on a weakly reflective substrate such as CaF_2_. In contrast, for very thin layers, the proximity of the Au substrate forces the field lines to align vertically, preventing the coupling to in-plane molecular vibrations.

To study the evolution of the near-field spectroscopic contrast with varying layer thickness *d* in more detail, we define the spectroscopic amplitude contrast Δ*s*_2_ as the difference between the maximum and minimum values in the amplitude spectra *s*_2_(ν). Similarly, we define the spectroscopic phase contrast Δφ_2_ as the peak height in the phase spectra φ_2_(ν). Figure 4a,b shows Δ*s*_2_ and Δφ_2_ for layers on a CaF_2_ substrate. We find that both amplitude and phase spectroscopic contrasts decrease for all three types of layers (isotropic, with in-plane vibrations and with out-of-plane vibrations) as the layer thickness *d* decreases, due to the reduced amount of absorbing material.^17,26,47^ Furthermore, for all layer thicknesses, the isotropic layers exhibit a higher spectroscopic contrast. This can be explained by the structure of the dielectric tensors ε⃡_iso_(ν), ε⃡_in_(ν) and ε⃡_out_(ν) that characterize the molecular vibrations of the layers. Specifically, Δ*s*_2_ and Δφ_2_ depend on the number of tensor components that contain the dielectric function ε­(ν), which represents the molecular oscillator. In the isotropic case, ε­(ν) appears in three components, while in the anisotropic cases, it appears in one or two components (see tensors in Figure 1d). For this reason, the total contribution of the molecular oscillators in the layer, and hence Δ*s*_2_ and Δφ_2_, is largest for the isotropic case, since each vibrational component is modeled using the same oscillator strength.

**Figure 4:**
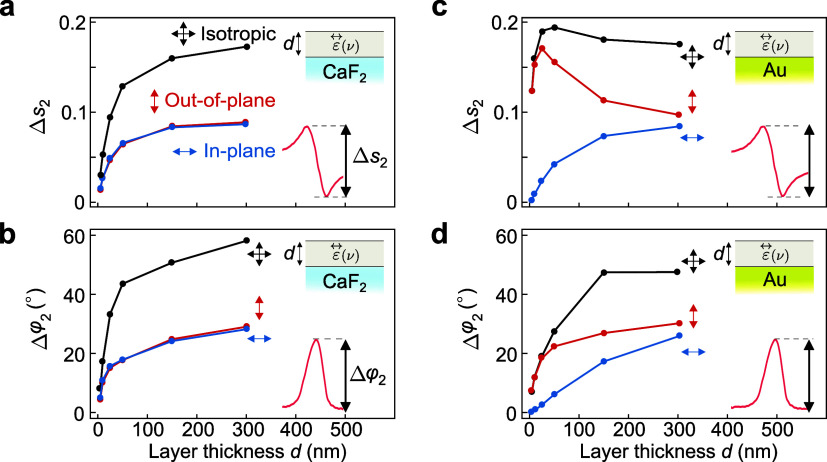
Thickness-dependent amplitude and phase contrast of near-field spectra. (a) Amplitude contrast Δ*s*_2_ and (b) phase contrast Δφ_2_ extracted from the calculated near-field spectra as a function of the layer thickness *d* for layers with ε⃡_iso_(ν) (black), ε⃡_in_(ν) (blue), and ε⃡_out_(ν) (red) on a CaF_2_ substrate. Insets in (a, b) from top to bottom: diagrams of the system and definitions of Δ*s*_2_ and Δφ_2_, respectively. (c, d) Same as in (a and b), but for layers on an Au substrate.

Notably, Δ*s*_2_ and Δφ_2_ are similar for the in-plane and out-of-plane anisotropic layers (blue and red curves), although the layers with in-plane vibrations include ε­(ν), i.e., molecular oscillators, in twice as many tensor components as the layers with out-of-plane vibrations. This finding can be explained by the stronger vertical component of the near fields generated by the tip. As observed in Figure 2g, the field lines are tilted by more than 45° relative to the sample surface, which leads to stronger coupling with out-of-plane vibrations as compared to in-plane vibrations.

For layers on the Au substrate, the evolution of the spectroscopic amplitude contrast Δ*s*_2_ with layer thickness *d* varies significantly depending on the molecular vibration orientation (see Figure 4c). For layers with in-plane vibrations (blue), reducing *d* decreases Δ*s*_2_. Conversely, for isotropic layers (black) and for layers with out-of-plane vibrations (red), reducing *d* increases Δ*s*_2_, reaching a maximum value at a thickness of approximately *d* ∼ 50 and *d* ∼ 25 nm, respectively, before decreasing for thinner layers. Comparing the contrasts Δ*s*_2_ for thin isotropic layers and thin layers with out-of-plane vibrations on the Au substrate (Figure 4c) to the corresponding layers on the CaF_2_ substrate (Figure 4a), we find a significant enhancement in Δ*s*_2_. This enhancement can be explained by the stronger tip–substrate near-field coupling on Au compared to that on CaF_2_.^47^ However, the near-field coupling between tip and Au substrate causes a more vertical orientation of the near fields close to the Au substrate, suppressing Δ*s*_2_ for thin layers with in-plane vibrations.^31^ We also find that the spectroscopic phase contrast Δφ_2_ evolves differently with thickness *d* for the three types of layers, and furthermore, it deviates from the behavior of the layers on the CaF_2_ substrate. For thin layers with out-of-plane vibrations on the Au substrate (red curve in Figure 4d), Δφ_2_ is notably larger than on a CaF_2_ substrate (red curve in Figure 4b), whereas the opposite behavior is found for thin layers with in-plane vibrations. This behavior can be understood analogously to that of the amplitude contrasts Δ*s*_2_.

In Figure 5 we discuss the spectral shifts of the molecular vibrational features described in Figures 2 and 3. To that end, we extract the spectral position of the phase peak, ν_φ2_^max^, from the near-field phase spectra and plot it as a function of the layer thickness *d*. Figure 5a shows ν_φ2_^max^ for layers on a CaF_2_ substrate. As the layer thickness *d* increases, we observe a blueshift of the peak position for both isotropic layers (black) and layers with in-plane vibrations (blue), whereas the peak positions of the layers with out-of-plane vibrations (red) exhibit a slight redshift. Notably, for thicker layers, reaching the bulk limit, ν_φ2_^max^ assumes different values for isotropic and anisotropic layers. Further, all peak positions are blue-shifted relative to the molecular vibration resonance frequency at ν = 1730 cm^–1^ (dashed-gray line), which has been observed in experimental near-field spectral of isotropic molecular layers.^16,17^ A similar behavior is observed for the layers on the Au substrate (Figure 5b).

**Figure 5:**
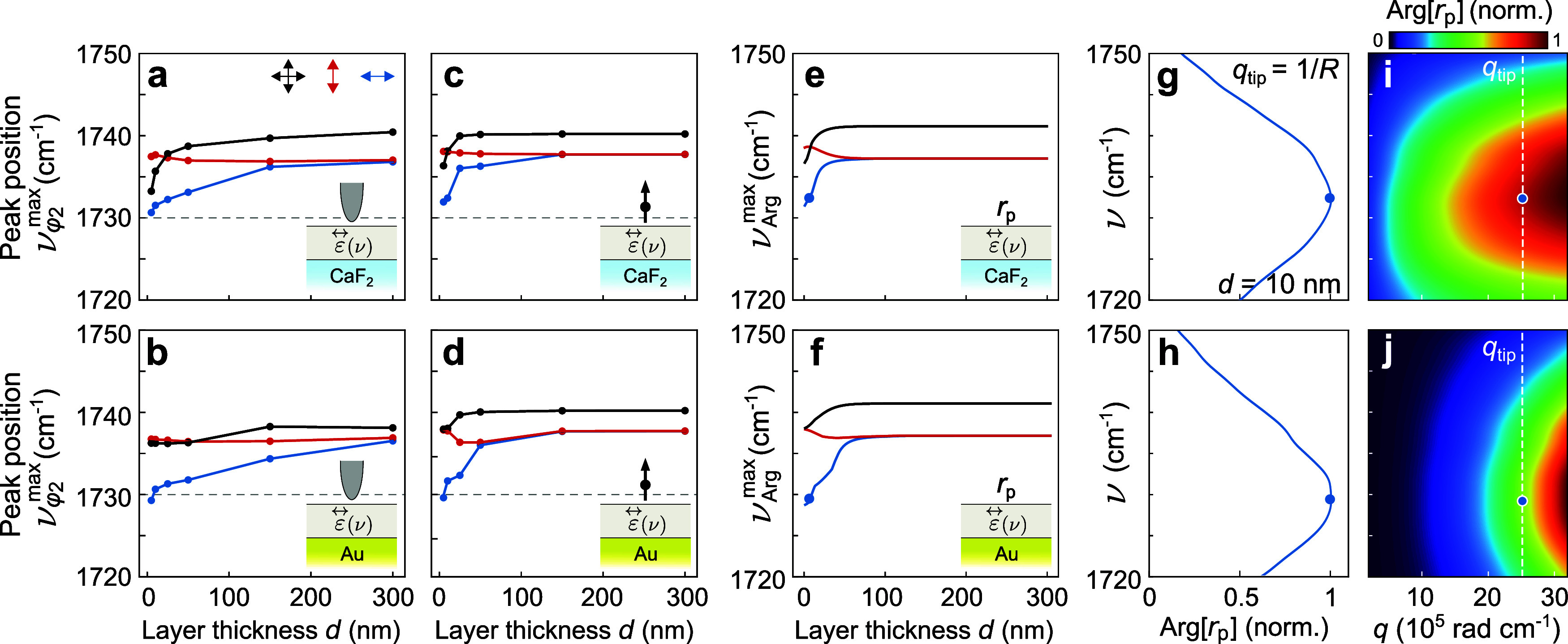
Thickness-dependent spectral shifts in near-field phase spectra. (a, b) Spectral peak position ν_φ2_^max^ extracted from calculated near-field phase spectra as a function of layer thickness *d*, for layers with ε⃡_iso_(ν) (black), ε⃡_in_(ν) (blue) and ε⃡_out_(ν) (red) dielectric tensors on a CaF_2_ substrate and on an Au substrate, respectively. (c, d) Spectral peak position ν_φ2_^max^ extracted from the near-field phase spectra obt**a**ine**d** from the ^eu‑^PDM for the same anisotropic layers. In (a–d), the insets represent configuration adopted in the calculations, and the horizontal dashed line indicates the molecular vibration frequency around ν = 1730 cm^–1^. (e, f) Spectral peak position ν_Arg_^max^ of the phase of the electrodynamic reflection coefficient, Arg­[*r*_p_(*q*_tip_,ν)], plotted as a function of the layer thickness *d* for the same sample configurations as in (a, c) and (b, d). This analysis assumes a p-polarized electromagnetic field with in-plane wavevector *q*_tip_ = 1/*R*, where *R* = 25 nm. (g, h) Frequency ν versus normalized Arg­[*r*_p_(*q*_tip_,ν)] for a 10 nm-thin layer with ε⃡_in_(ν)­on CaF_2_ and on Au, respectively. (i, j) Arg­[*r*_p_(*q*,ν)] for the same 10 nm-thin layer, plotted as a function of the in-plane wavevector *q* and the wavenumber ν of the reflected p-polarized wave. The white dashed line marks *q*_tip_. Blue dots in (e–j) mark the frequency ν_Arg_^max^, where Arg­[*r*_p_(*q*_tip_,ν)] exhibits its maximum.

To better understand the peak shifts observed in the near-field spectra obtained by our electrostatic numerical calculations and to elucidate their origin, we develop an analytical electrodynamic model. Analogous to a previously developed electrodynamic point-dipole model,^26^ the tip is described as a point dipole with a polarizability corresponding to that of a particle with radius equal to the tip apex radius. We expand the previous isotropic model to be able to address uniaxial multilayer samples (details in SI section 4). We thus refer to this model as the Electrodynamic Point Dipole Model for Uniaxial Layers (^eu‑^PDM). Although this point-dipole model fails to quantitatively describe experimental near-field spectra because it neglects the elongated tip shape,^49^ it is fully electrodynamic and, importantly, relates the near-field spectra to Fresnel reflection coefficients *r*_p_. The near-field spectra calculated with this ^eu‑^PDM exhibit good qualitative agreement with the near-field spectra obtained with our electrostatic numerical calculations (shown in SI section 5). In Figure 5c,d we show ν_φ2_^max^ obtained from these spectra, corroborating the results from the electrostatic numerical calculations (in Figure 5a,b) for all three types of layers.

Following a previous study that identified the Fresnel reflection coefficient of the sample as the root cause of peak shifts in near-field spectra of isotropic layers,^17^ we analyze the multilayer reflection coefficient of the sample, *r*_p_(*q*,ν), where *q* is the in-plane wavevector and ν the wavenumber of the reflected p-polarized electromagnetic field. In Figure 5i,j, we show, as an example, Arg­[*r*_p_(*q*,ν)] for a 10 nm-thin layer with in-plane molecular vibrations on CaF_2_ and Au substrates. Since in the PDM the tip’s near fields predominantly probe the reflection coefficient at a wavevector *q*_tip_ = 1/*R*, where *R* is the radius of the tip apex,^2,21,50^ we determine the spectral peak position of Arg­[*r*_p_(*q*_tip_,ν)], as illustrated in Figure 5g,h for the reflection coefficient of the 10 nm-thin layers shown in Figure 5i,j. The spectral peak position is denoted ν_Arg_^max^ and marked in Figure 5g–j by blue dots. In Figure 5e,f, we plot ν_Arg_^max^ as a function of layer thickness *d* for layers on CaF_2_ and Au substrates. Comparison with the spectral peak shifts observed in the near-field phase spectra obtained from the ^eu‑^PDM calculations reveals good quantitative agreement. This confirms that the dependence of peak shifts on both the layer thickness and the orientation of molecular vibrations is primarily determined by the Fresnel reflection coefficient of the multilayer samples.

As shown above, the results obtained with the ^eu‑^PDM yield nearly the same peak positions as the numerical calculations, but they lack overall quantitative accuracy, as has also been observed with the electrostatic PDM.^19,21,49^ In contrast, numerical calculations are more precise but computationally demanding, making them impractical for large-scale studies. Therefore, we seek a calculation method that is both fast and accurate. The Finite Dipole Model (FDM) is known for offering both speed and reasonably good accuracy,^18,21,28,49^ however, it has not yet been developed for anisotropic thin layers. To model the near-field spectra of anisotropic thin layers within the FDM, we use the perturbative approach introduced in ref 18. This method is easier to implement and requires fewer empirical parameters as compared to other thin-layer FDMs.^21^ In the perturbative FDM (details in Methods), the near-field probe is modeled as a metallic ellipsoid, and the thin layer is treated as the difference of two virtual half-spaces.^18^ Since the original model does not inherently account for anisotropy, we implement an adaptation inspired by transformation optics.^51^ In this approach, the dielectric tensor components of the layer, ε_*xx*_ (ν), ε_*zz*_ (ν) and the layer thickness *d*, are transformed using a conformal mapping (derivation in Methods). This results in a new scalar dielectric function $\varepsilon^{\prime }=\sqrt{\varepsilon_{\mathrm{xx}}\left(\nu \right)\varepsilon_{\mathrm{zz}}\left(\nu \right)}$ and virtual layer thickness $d^{\prime }=d\sqrt{\varepsilon_{\mathrm{xx}}\left(\nu \right)/\varepsilon_{\mathrm{zz}}\left(\nu \right)}$ , which allows the standard perturbative FDM to be applied to uniaxial samples. Note that *d*′ can be a complex number, whose imaginary part must be also considered for the calculations.^52^ We also note that the application of the perturbative FDM is limited to molecular vibrations (weak oscillators) and is not suitable for modeling layers comprising strong oscillators (phonons), i.e., where Re­[ε­(ν)]<0.

In Figure 6 we compare the near-field phase spectra for 25 nm-thin layers on CaF_2_ and Au substrates, calculated using numerical electrostatic simulations, ^eu‑^PDM and perturbative FDM. While the spectra obtained with the ^eu‑^PDM (dashed lines) exhibit spectral peak positions similar to those of the numerically calculated spectra (dots), the peak heights are largely overestimated, particularly in Figure 6b–d,f. Conversely, the spectra obtained with the perturbative FDM (solid lines) reproduce well both the peak positions and peak heights of the numerically calculated spectra, as can be observed in all panels of Figure 6. As shown in the SI sections 5 and 6, the perturbative FDM agrees well with the numerical calculations for layer thicknesses between 10 and 150 nm. For thinner layers, discrepancies with the numerical calculations arise, due to neglecting third order and higher-order terms in the perturbative expansion. For thicker layers, the accuracy of the perturbative FDM is reduced, which we attribute to limitations of the underlying bulk FDM itself. Overall, the results demonstrate that the perturbative FDM is an effective method for calculating near-field spectra of thin uniaxial organic layers on different substrates, achieving accuracy close to that of numerical calculations while being significantly faster (few seconds compared to several hours for each spectrum).

**Figure 6:**
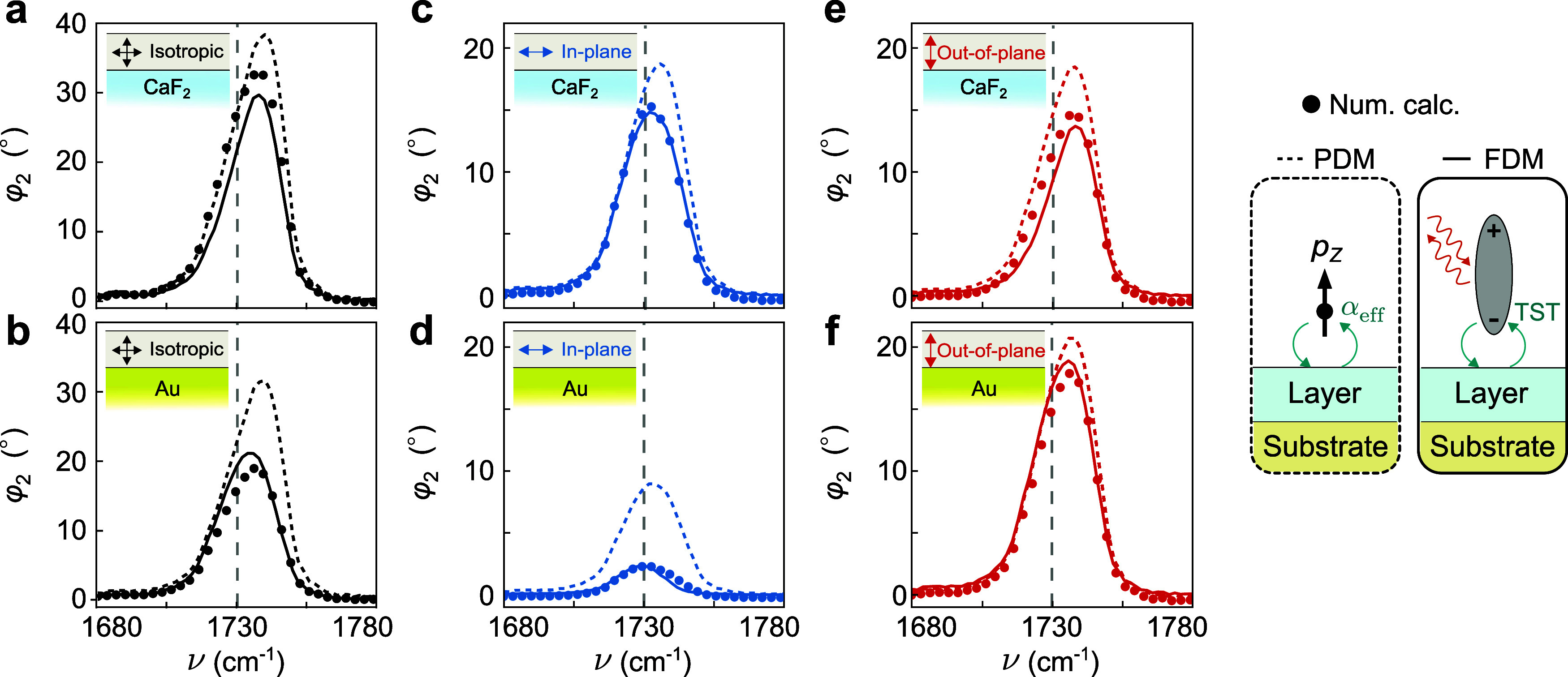
Comparison of the near-field phase spectra obtained by the numerical electrostatic calculations, the ^eu‑^PDM and the perturbative FDM for uniaxial layers. (a) Calculated near-field phase spectra for 25 nm-thin layers with ε⃡_iso_(ν) on a CaF_2_ substrate by the numerical electrostatic calculations (black dots); the ^eu‑^PDM (black-dashed line); and the perturbative FDM for uniaxial layers (black-solid line). (b) Same as (a) for 25 nm-thin layers with ε⃡_iso_(ν) on an Au substrate. (c–f) Same as in (a, b), but for layers with ε⃡_in_(ν) and ε⃡_out_(ν), respectively. Insets in (a–f) represent the molecular orientation in the layer and the substrate configuration.

We finally note that throughout our study we have focused on the analysis of amplitude *s*_2_(ν) and phase φ_2_(ν) spectra, with the latter providing information about the absorption in the sample. As an alternative to phase, the imaginary part of the complex-valued near-field spectrum, σ_2_(ν) = *s*_2_(ν)*e* ^*i*φ_2_(ν)^, has been introduced as a potentially more physical description of absorption.^10,16,19,41^ This approach is motivated by the principle that the absorption in any small object with polarizability α (e.g., the spherical particle approximating the tip in the PDM) is proportional to Im­[α] ^6,53,54^ rather than Arg­[α]. We provide a comparison of both quantities in SI section 7. In brief, we find that both φ_2_- and Im­[σ_2_]-spectra are valid for analyzing molecular vibrational absorption in thin layers, but peak positions may differ from those observed in far-field infrared spectroscopies.^16,17,19,31,55^ Accurate peak identification or precise comparison with infrared spectra from databases may thus require reconstructing the sample′s dielectric function from the near-field spectra through modeling. The dielectric function can be used to calculate the corresponding far-field spectra, which then can be directly compared with database spectra for reliable interpretation of spectral peaks. As an example of the differences that arise from using φ_2_- or Im­[σ_2_]-spectra, we find that the spectral contrast of Im­[σ_2_]-spectra for both isotropic layers and layers with out-of-plane vibrations on Au substrates increase nonmonotonically with decreasing layer thickness,^16^ unlike that of φ_2_-spectra.^17,26,56^ This phenomenon arises from the increasing near-field coupling between the tip and the Au substrate,^26,47^ which contributes to Im­[σ_2_(ν)] and thus complicates the interpretation of molecular vibrational absorption compared to the analysis based on phase spectra. Consequently, we consider φ_2_-spectra to be more straightforward to interpret, especially when theoretical modeling is not feasible, e.g., when the substrate effects or other unknown parameters complicate the analysis.

## Conclusions

In summary, we systematically studied the infrared near-field spectra of uniaxial thin layers with molecular vibrations of different orientations using numerical electrostatic calculations, supported by an electrodynamic PDM. Our analysis demonstrates that elongated near-field probes are sensitive to in-plane vibrations in thick layers and thin layers on weakly reflecting substrates, such as CaF_2_. However, experimental near-field signals of thin layers on such substrates are typically low, which may challenge the detection of molecular vibrations. When thin layers are placed on highly reflective substrates, such as metals or Si, the near-field signal is generally enhanced, but the sensitivity to in-plane vibrations is strongly reduced. This effect is not due to the elongated shape of the probe but rather to the vertical orientation of the near fields with respect to the layer, determined by electromagnetic boundary conditions at the surface of highly reflecting substrates. Furthermore, the calculated near-field spectra reveal that the spectral positions of molecular vibrational features depend on the orientation of the vibrations, layer thickness, and substrate, which can be explained by the behavior of the Fresnel reflection coefficients at high near-field wavevectors.

Numerical calculations provide detailed near-field spectra but are computationally demanding and time-consuming. The PDM, in contrast, is computationally efficient and offers valuable support for interpreting the numerical results by providing fundamental physical insights. However, it does not yield quantitative agreement with the numerical spectra. To develop a model that combines quantitative accuracy with practical computational efficiency, we extended the perturbative FDM to calculate infrared near-field spectra of uniaxial thin-layer samples. We demonstrated that this model accurately reproduces the numerically calculated near-field spectra for all the samples considered (isotropic layers, layers with in-plane vibrations and layers with out-of-plane vibrations) for a range of layer thicknesses between 10 and 150 nm, while providing results much faster (second time scale) than the numerical calculations. The faster and accurate performance of this model makes it a promising candidate for the inversion of near-field signals to reconstruct the dielectric permittivity of uniaxial samples, a direction for further investigation.

Our comprehensive study is of both fundamental and practical importance for analyzing, interpreting, and distinguishing molecular vibrational absorption in thin uniaxial organic layers on different substrates. Future studies could extend the presented analysis to biaxial thin-layer samples or to explore the use of peak shifts for analyzing molecular vibration orientation in thin organic layers.

## Methods

### Dielectric Functions

We model the IR response of the materials considered in the electrostatic numerical calculations, the electrodynamic PDM and the perturbative FDM as follows. The dielectric function for the platinum (Pt) tip ε_tip_, corresponds to a Brendel and Bormann model from ref 57. For poly­(methyl methacrylate) (PMMA), the dielectric function ε­(ν) describes the molecular vibrations in the layer, based on the PMMA carbonyl (C=O) stretching mode near 1730 cm^–1^. We use a Gaussian-convoluted Drude-Lorentz model from ref 44. The dielectric function of the gold (Au) and silicon (Si) substrates are obtained by linearly interpolating the data provided in ref 58 and ref 46, respectively, while for the calcium fluoride (CaF_2_) substrate is adapted from a model in ref 59 as follows

1 $$\varepsilon_{\mathrm{Ca}F_{2}}\left(\nu \right)=\varepsilon_{\infty }+\frac{S_{1}\Omega_{1}^{2}\left(\Omega_{1}^{2}-\nu^{2}\right)}{{\left(\Omega_{1}^{2}-\nu^{2}\right)}^{2}+\gamma_{1}^{2}\nu^{2}}$$

with ε_∞_ = 2.02, *S*_1_ = 4.18, Ω_1_ = 272.74 cm^–1^ and γ_1_ = 260.19 cm^–1^.

### Electrostatic Numerical Calculations

Electrostatic numerical calculations are implemented in the AC/DC Module of COMSOL Multiphysics software.^42^ This module solves Maxwell’s equations based on the Finite Element Method (FEM). The tip is described by a semiellipsoid with semiaxes $a=b=\sqrt{R\cdot L}$ and *c = L*, where *R* = 25 nm is the tip radius and *L* = 600 nm is the tip length. The multilayer system—comprising the environment (region where the tip is located), the anisotropic layer and the substrate—is modeled as rectangular boxes of lateral size *w*_b_ = 1800 nm and thicknesses *d*_env_, *d*, and *d*_sub_ (see Figure 1a). The tip and the multilayer are located inside a rectangular box (which simulates the universe) of lateral and vertical dimensions *w*_u_ = 2000 nm and *d*_u_ = 2800 nm. The incident electric field in the environment region *E⃗*_inc_ = *E*_inc_ *ẑ* (*E*_inc_ = 1 V/m) corresponds to a potential difference *V*_inc_ between the top and bottom layers in the simulation box. *V*_inc_ is the solution of Poisson’s equation in the multilayer system

$$V_{\mathrm{inc}}\left(\nu \right)=\left(\frac{\varepsilon_{\mathrm{env}}\left(\nu \right)}{\varepsilon_{\mathrm{sub}}\left(\nu \right)}d_{\mathrm{sub}}+\frac{\varepsilon_{\mathrm{env}}\left(\nu \right)}{\varepsilon_{\mathrm{zz}}\left(\nu \right)}d+d_{\mathrm{env}}\right)E_{\mathrm{inc}}$$ 2

where ε_env_ = ε_0_ = 1 and ε_sub_ are the dielectric functions of the environment and the substrate, respectively. ε_*zz*_ is the out-of-plane component of the dielectric tensor for the anisotropic layer.

The geometric regions in the calculations are meshed as follows. Tip: Free Tetrahedral, Automatic tessellation (AT), Max element size MES = 15 nm, minimum element size mES = 0.8 nm, curvature factor CF = 0.15. Layer surface: Free triangular, Delaunay Tessellation (DT), MES = 30 nm. Layer volume: Linear Swept of the layer surface, Distribution elements DE = 20. Substrate volume: Linear Swept of the layer surface, DE = 10. Remaining regions: Predefined mesh, Extra fine size.

To calculate the demodulated near-field spectra using electrostatic numerical calculations, we assume that the tip-scattered field is *E*_sca_(*h*,ν) ∝ *p*(*h*,ν), where *p*(*h*,ν) ∼ *p*_*z*_(*h*,ν) is the total and nearly vertical dipole moment induced in the tip at a fixed tip–sample distance *h*. The vertical dipole moment *p*_*z*_ is defined as the first moment in the charge distribution along the tip surface *S*_tip_, expressed as

3 $$p_{z}\left(h,\nu \right)=\varepsilon_{0}\left(1-\frac{1}{\varepsilon_{\mathrm{tip}}\left(\nu \right)}\right)\oint_{S_{\mathrm{tip}}}z\left[\mathrm{E⃗}\left({\mathrm{r⃗}}^{\prime }+hẑ\right)\cdot {û}_{n}\right]d^{2}r^{\prime }$$

where *û*_*n*_ is the outward normal vector of the surface *S*_tip_. The tip–sample distance *h* varies periodically with time *t* as

4 $$h\left(t\right)=h_{0}+A\left[1-\cos\left(\Omega t\right)\right]$$

mimicking a vertical oscillation with amplitude *A*, frequency Ω and period *T* = 2π/Ω. We choose a set of tip–sample distances determined by *h* = *h*_0_+10^*e*_*m*_^, where *h*_0_ is the minimum tip–sample distance (*h*_0_ = 1 nm) and *e*_*m*_ is

5 $$e_{m}=\log_{10}{(h}_{0})+\frac{m-1}{N-1}\left[\log_{10}{(2A+h}_{0})-\log_{10}{(h}_{0})\right]$$

where *m* = 1, 2, ···, *N*, and *A* = 50 nm. *N* is the total number of heights considered for the calculations. We typically choose *N* = 15. The demodulated signal is obtained by calculating the *n-*th Fourier coefficient of *p*_*z*_(*h*(*t*),ν) as

6 $$\sigma_{s,n}\left(\nu \right)=s_{s,n}\left(\nu \right)e^{i\varphi_{s,n}\left(\nu \right)}\propto \int_{0}^{T}e^{\mathrm{in}\Omega t}p_{z}\left(h\left(t\right),\nu \right)dt$$

The coefficients σ_*s,n*_(ν) are computed using the fast Fourier transform (FFT) algorithm from the SciPy library in Python.

### PDM Calculations

Electrodynamic point-dipole simulations for uniaxial layers are obtained by modeling the tip as an electric point dipole with polarizability α_tip_(ν), located in air at a distance *z*_0_ above the multilayer sample. By placing the point dipole in close proximity to the multilayer sample, the near-field interaction between the point dipole and the sample polarizes the dipole, resulting in a net vertical dipole moment *p*_*z*_. Within this framework, we interpret the tip-scattered field as the radiation emitted by the net vertical dipole moment, which we calculate as in ref 26

$$p_{z}\left(z_{0},\nu \right)=\alpha_{\mathrm{eff}}\left(z_{0},\nu \right)E_{\mathrm{inc}}$$ 7

where

$$\alpha_{\mathrm{eff}}\left(z_{0},\nu \right)=\frac{\alpha_{\mathrm{tip}}\left(\nu \right)}{1-\omega^{2}\mu_{0}\alpha_{\mathrm{tip}}\left(\nu \right)G_{\mathrm{zz}}\left({\mathrm{r⃗}}_{0},{\mathrm{r⃗}}_{0},\nu \right)}$$ 8

is the effective polarizability of the point dipole, evaluated at its position *r⃗*_0_ = (*z*_0_,0,0). μ_0_ is the permeability of free space and *G*_*zz*_(*r⃗*_0_,*r⃗*_0_,ν) is the self-interaction Green’s function for the multilayer system determined by the following expression

$$G_{\mathrm{zz}}\left({\mathrm{r⃗}}_{0},{\mathrm{r⃗}}_{0},\nu \right)=\frac{i}{4\pi k_{0}^{2}}\int_{0}^{\infty }\frac{q^{3}}{k_{1z}}e^{i2k_{1z}z_{0}}r_{p}dq$$ 9

with *k*_1*z*_^2^ = *k*_0_^2^ *q* ^2^, *k*_0_ = ω/*c* (*c* is the speed of light in free space) and *q* is the transverse component of the wavevector. Further details of the ^eu‑^PDM and a step-by-step derivation of eqs 7–9 are provided in the SI.

To calculate the demodulated near-field spectra using the ^eu‑^PDM, we employ a similar procedure to that used for electrostatic calculations. Specifically, we substitute eq 4 into eqs 7–9 and compute the net vertical dipole moment *p*_*z*_(*h*(*t*)) over a single period, ranging from 0 to *T* = 2π/Ω. The calculation is performed for 200 discrete points, uniformly spaced across the interval. We use the same values for the parameter *h*_0_, *A* and Ω as those used in the electrostatic calculations. With the list of *p*_*z*_ values for different tip heights, we obtain σ_*s,n*_(ν) as the *n*-th Fourier coefficient obtained by Fourier transforming the list of values using the Fourier package of Wolfram Mathematica software.

### FDM Calculations for Bulk Samples

We applied and adapted a perturbative finite dipole model (FDM) for thin molecular films on a substrate.^18^ This model is based on the standard theoretical description of s-SNOM using the FDM for bulk samples.^49^ In detail, the light scattering from the tip is described by the scattering coefficient, σ = |*E*_sca_|/|*E*_inc_|, which relates the tip-scattered field, *E*_sca_, to the incident field *E*_inc_. The incident field polarizes the tip, which yields an effective dipole moment *p* = α_eff_(1 + *r*)*E*_inc_, where α_eff_ is the effective polarizability of the tip that accounts for the near-field coupling between the tip and the sample. *r* is the far-field reflection coefficient of the sample surface. The field backscattered by the tip can be subsequently described as *E*_sca_ = (1 + *r*)*p*. Thus, the scattering coefficient can be written as

10 $$\sigma =\alpha_{\mathrm{eff}}{\left(1+r\right)}^{2}$$

A solution for the effective tip polarizability α_eff_ can be obtained in the electrostatic approximation, which relates α_eff_ with the sample dielectric permittivity, ε

11 $$\alpha_{\mathrm{eff}}=C\left(1+\frac{1}{2}\cdot \frac{f_{0}\left(h\right)\beta \left(\varepsilon \right)}{1-f\left(h\right)\beta \left(\varepsilon \right)}\right)$$

where *f*_0_(*h*) and *f­(h)* are model-specific functions describing the effect of the tip–sample distance, *h*, and β­(ε) = (ε – 1)/(ε + 1) is the quasistatic reflection coefficient the semi-infinite half-space made of the sample material and *C* is a height-independent constant.

### Perturbative FDM for Isotropic Layers

The original perturbative FDM for thin films considers isotropic thin films of thickness *d* on a substrate. To this end, the tip–sample interaction is described in form of multiple scattering events between the tip, the thin film and the substrate. Each individual scattering event considers that the tip is interacting via the near field with a virtual half-space occupied by either the thin film material (ε) or the substrate material (ε_sub_), beginning at *z* = 0 or *z* = −*d*. Specifically, the following three first order interaction terms are obtained: (i) the scattering tensor, *T⃡*_sca_(ε_sub_,*d*), describing the scattering off the substrate at *z* = −*d*, (ii) the scattering tensor, *T⃡*_sca_(ε,0), describing the scattering off virtual half-space occupied by thin film material at *z* = 0, and (iii) the scattering tensor, *T⃡*_sca_(ε,*d*), describing the scattering off virtual half-space occupied by thin film material at *z* = −*d*. Summation over the individual scattering events in form of a Born series expansion yields the total near-field interaction of the tip with a thin film on a substrate (see also Figure 3 in ref 18)­

$${\mathrm{T⃡}}_{\mathrm{sca}}={\mathrm{T⃡}}_{\mathrm{sca}}\left(\varepsilon_{\mathrm{sub}},d\right)+{\mathrm{T⃡}}_{\mathrm{sca}}\left(\varepsilon ,0\right)-{\mathrm{T⃡}}_{\mathrm{sca}}\left(\varepsilon ,d\right)+\mathrm{SO}+\mathrm{HO}$$ 12

The original perturbative FDM for thin films considered terms up to second interaction order, SO, and truncated higher order terms, HO, for practical purposes. The resulting effective polarizability is then expressed as a sum of a total of 11 terms

$$\begin{array}{l} \alpha_{\mathrm{eff}}=C[2+\xi_{0}\left(\varepsilon_{\mathrm{sub}},d\right)+\xi_{0}\left(\varepsilon ,0\right)-\xi_{0}\left(\varepsilon ,d\right)+2\xi_{1}\left(\varepsilon ,d\right)\xi_{0}\left(\varepsilon ,d\right)\\ \begin{array}{l} -\left(\xi_{1}\left(\varepsilon ,0\right)\xi_{0}\left(\varepsilon ,d\right)+\xi_{1}\left(\varepsilon ,d\right)\xi_{0}\left(\varepsilon ,0\right)\right)\\ +\left(\xi_{1}\left(\varepsilon ,0\right)\xi_{0}\left(\varepsilon_{\mathrm{sub}},d\right)+\xi_{1}\left(\varepsilon_{\mathrm{sub}},d\right)\xi_{0}\left(\varepsilon ,0\right)\right) \end{array}\\ -\left(\xi_{1}\left(\varepsilon ,d\right)\xi_{0}\left(\varepsilon_{\mathrm{sub}},d\right)+\xi_{1}\left(\varepsilon_{\mathrm{sub}},d\right)\xi_{0}\left(\varepsilon ,d\right)\right)] \end{array}$$ 13

with

$$\begin{array}{l} \xi_{0}\left(\varepsilon ,d\right)=f_{0}\left(h+d\right)\beta \left(\varepsilon \right){\left[1-f\left(h+d\right)\beta \left(\varepsilon \right)\right]}^{-1}\\ \xi_{1}\left(\varepsilon ,d\right)=f\left(h+d\right)\beta \left(\varepsilon \right){\left[1-f\left(h+d\right)\beta \left(\varepsilon \right)\right]}^{-1} \end{array}$$ 14

and

$$\begin{array}{l} f_{0}\left(h\right)=\left(g-\frac{2h+W_{0}+R}{2L}\right)\frac{\ln\frac{4L}{4h+2W_{0}+R}}{\ln\frac{4L}{R}}\\ f\left(h\right)=\left(g-\frac{2h+W_{i}+R}{2L}\right)\frac{\ln\frac{4L}{4h+2W_{i}+R}}{\ln\frac{4L}{R}} \end{array}$$ 15

where we set *W*_0_ ≈ 1.31*R* and *W*_i_ ≈ 0 with *R* being the tip radius. For the two model parameters we choose the values:^49^ *L* = 300 nm is the semiaxis of the ellipsoid modeling the tip, and *g* = 0.7. Vertical tip oscillation, *h* = *h*(*t*), at frequency Ω and subsequent signal demodulation at a higher harmonic, *n*Ω, are considered by calculation of the *n*-th Fourier coefficient of the scattering coefficient

16 $$\sigma_{n}\left(\beta \right)=F_{n}\left[\sigma \left(\beta ,h\left(t\right)\right)\right]=\int_{0}^{T}\sigma \left(\beta ,h\left(t\right)\right)e^{\mathrm{in}\Omega t}dt$$

This quantity, σ_*n*_(β), is proportional to the *n*-th order demodulated detector signal in the experiment. The constant *C* ∝ *W*_0_^2^ *E*_inc_ cancels out after normalizing the signal to that of a reference material.

To calculate the demodulated signal, we compute α_eff_(*h*(*t*),ν) over a single tip-vertical oscillation period (see eq 4), ranging from 0 to *T* = 2π/Ω. The calculation is performed for 50 discrete points, uniformly spaced across the time interval. Then the Fast Fourier Transform of α_eff_(*h*(*t*),ν) is performed for each linear frequency ν in Matlab software.

### Perturbative FDM for Uniaxial Layers

The above model uses the scalar description for the thin film dielectric permittivity to describe isotropic thin films. Within this limitation, we introduce the following modification of this model to consider uniaxial thin layers on a substrate. This change is motivated by transformation optics approach as discussed below. Specifically, the following modifications are introduced for the layer thickness, $d^{\prime }=d\sqrt{\varepsilon_{\mathrm{xx}}\left(\nu \right)/\varepsilon_{\mathrm{zz}}\left(\nu \right)}$ , and the thin film dielectric permittivity, $\varepsilon^{\prime }=\sqrt{\varepsilon_{\mathrm{xx}}\left(\nu \right)\varepsilon_{\mathrm{zz}}\left(\nu \right)}$ where ε_*xx*_(ν), ε_*zz*_(ν) are the in-plane and out-of-plane dielectric tensor components, respectively. Note that the virtual layer thickness, *d*′, becomes a complex-valued quantity.

### Description of Anisotropic Layers Using Transformation Optics

Since the perturbative FDM cannot account for anisotropy in the layer, we implement a procedure inspired by transformation optics. Transformation optics is a theoretical framework that exploits the coordinate invariance of Maxwell’s equations.^51,60^ It enables to redesign the spatial coordinates where electromagnetic waves propagate, as if they were propagating through a transformed medium with different optical properties. In our case, we use transformation optics to represent the uniaxial layer, described by the dielectric tensor

17 $$\mathrm{\varepsilon ⃡}=\left[\begin{array}{lll} \varepsilon_{\mathrm{xx}} & 0 & 0\\ 0 & \varepsilon_{\mathrm{xx}} & 0\\ 0 & 0 & \varepsilon_{\mathrm{zz}} \end{array}\right]$$

as an effective isotropic layer with dielectric function ε′. To find such re-expression, we consider that there may exist a coordinate transformation described by the tensor *M⃡*, such that the material dielectric properties can be described through the scalar ε′ in the transformed coordinate system. Specifically, *M⃡* needs to satisfy the condition

18 $$\varepsilon^{\prime }\mathrm{I⃡}=\left[\begin{array}{lll} \varepsilon^{\prime } & 0 & 0\\ 0 & \varepsilon^{\prime } & 0\\ 0 & 0 & \varepsilon^{\prime } \end{array}\right]=\frac{\mathrm{M⃡}\mathrm{\varepsilon ⃡}{\mathrm{M⃡}}^{T}}{\left|\mathrm{M⃡}\right|}$$

Since both ε′I⃡ and ε⃡ are diagonal tensors, we simplify the problem by seeking a diagonal form for *M⃡*

19 $$\mathrm{M⃡}=\left[\begin{array}{lll} M_{11} & 0 & 0\\ 0 & M_{22} & 0\\ 0 & 0 & M_{33} \end{array}\right]$$

From eq 18, we obtain the linear system with unknowns *M*_11_, *M*_22_, *M*_33_ and parameter ε′

20 $$\begin{array}{l} \varepsilon^{\prime }=\frac{M_{11}\cdot \varepsilon_{\mathrm{xx}}}{M_{22}\cdot M_{33}}\\ \varepsilon^{\prime }=\frac{M_{22}\cdot \varepsilon_{\mathrm{xx}}}{M_{11}\cdot M_{33}}\\ \varepsilon^{\prime }=\frac{M_{33}\cdot \varepsilon_{\mathrm{zz}}}{M_{11}\cdot M_{22}} \end{array}$$

Solving this system requires that *M*_11_^2^ = *M*_22_^2^ and *M*_33_^2^ = (ε_*xx*_/ε_*zz*_)*M*_11_^2^. A possible solution is *M*_11_ = *M*_22_ = 1, and $M_{33}=\sqrt{\varepsilon_{\mathrm{xx}}/\varepsilon_{\mathrm{zz}}}$ . With this solution, the effective scalar dielectric function becomes $\varepsilon^{\prime }=\sqrt{\varepsilon_{\mathrm{xx}}\varepsilon_{\mathrm{zz}}}$ . The corresponding coordinate transformation to the new system *(x*′, *y*′, *z′*) is

21 $$\left(x^{\prime },y^{\prime },z^{\prime }\right)=\mathrm{M⃡}\left(x,y,z\right)=\left(M_{11}x,M_{22}y,M_{33}z\right)$$

Since *M*_11_=*M*_22_ = 1, the *x-* and *y*-directions remain unchanged in the new system, whereas only the vertical *z*-direction is rescaled. Specifically, if the anisotropic material forms a layer of thickness *d*, its thickness in the transformed coordinate system becomes $d^{\prime }=d\sqrt{\varepsilon_{\mathrm{xx}}/\varepsilon_{\mathrm{zz}}}$ .

## Supplementary Material



## Data Availability

The data and complementary information about the numerical calculations and theoretical models used in this study are openly available in the Zenodo repository: 10.5281/zenodo.15480863

## Notes

Detailed comparisons of electrodynamic and electrostatic numerical calculation methods, comparison of near-field spectra for thin anisotropic layers on different substrates, analysis of electric field lines in thin anisotropic layers, derivation of the analytical model describing the tip as an electric point dipole (^eu‑^PDM), a complete collection of calculated near-field spectra with different methods, comparison of spectroscopic amplitude and phase contrasts, and investigation of the relationship between phase and the imaginary part of near-field spectra (PDF)
