# Supplementary material for: Theoretical Description of Infrared Near-Field Spectroscopy of In- and Out-of-Plane Molecular Vibrations in Thin Layers
Source: ACS Photonics. 2025 Jun 27;12(7):3782–93. doi: 10.1021/acsphotonics.5c00798 (PMC12272971; doi:10.1021/acsphotonics.5c00798)
Supplement: Supplementary file 1 [file ph5c00798_si_001.pdf]

Supporting Information for:

Theoretical Description of Infrared Near-Field Spectroscopy of In- and Out-of-Plane Vibrations in Thin Layers

Isabel Pascual Robledo[1,2], Carlos Maciel-Escudero[2], Martin Schnell[2,3,4], Lars Mester[5], Javier Aizpurua[3,4,6*] and Rainer Hillenbrand[2,4,6*]

[1]*Material Physics Center, CSIC-UPV/EHU, Paseo de Manuel Lardizabal 5, Donostia-San Sebastian 20018, Spain*
[2]*CIC nanoGUNE BRTA, Tolosa Hiribidea, Donostia-San Sebastian 20018, Spain*
[3]*Donostia International Physics Center, Paseo de Manuel Lardizabal 3, Donostia-San Sebastian 20018, Spain*
[4]*IKERBASQUE, Basque Foundation of Science, Bilbao 48011, Spain*
[5]*Attocube Systems GmbH, Eglfinger Weg 2, 85540 Haar, Germany*
[6]*Dept. of Electricity and Electronics, University of the Basque Country (UPV/EHU), Leioa, Spain*

*E-mail: r.hillenbrand@nanogune.eu
*E-mail: aizpurua@ehu.eus

Table of Contents

Section 1: Comparison between electrodynamic and electrostatic numerical calculations... 3

Section 2: Comparison of near-field spectra of thin layers on different substrates 4

Section 3: Electric field lines for thin anisotropic layers.. 5

Section 4: Numerical calculations where the tip is modeled as an electric-point dipole........ 5

Section 5: Collection of calculated near-field spectra.. 11

Section 6: Comparison of spectroscopic near-field amplitude and phase contrasts............ 12

Section 7: Phase *vs.* imaginary part of near-field spectra.. 12

Bibliography of the Supporting Information 14

Section 1: Comparison between electrodynamic and electrostatic numerical calculations

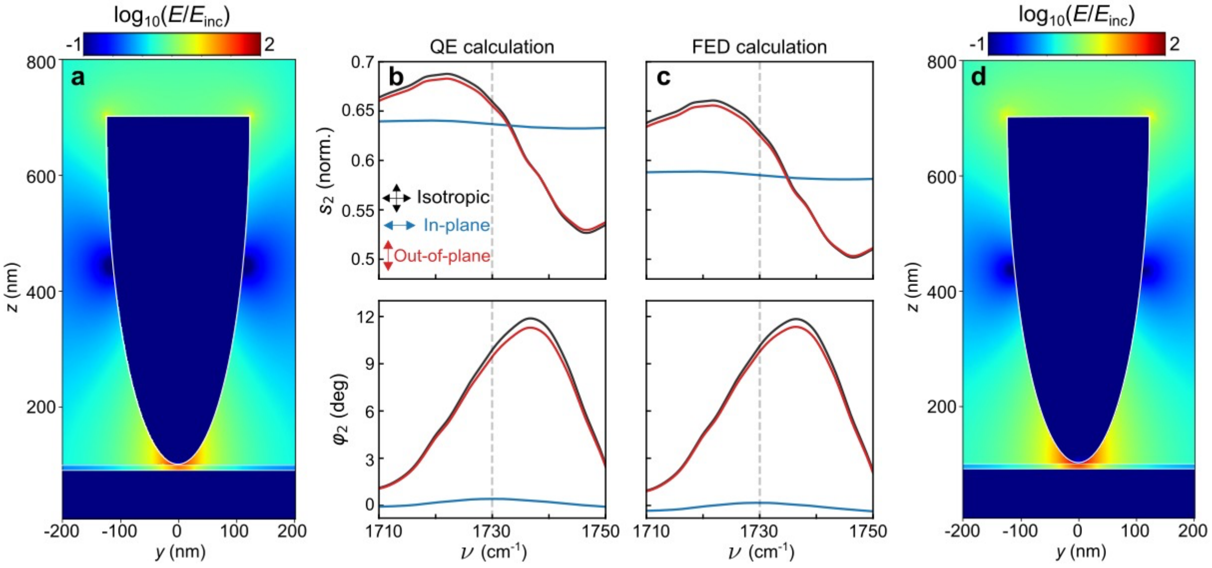

Figure S1. Comparison of Electrostatic and Electrodynamic numerical calculations. a Electrostatic field distribution around the Pt tip over a 10 nm-thin layer with in-plane vibrations on Au substrate obtained with the AC/DC COMSOL Module. **b** Amplitude (top) and phase (bottom) of the near field spectra for isotropic (black), in-plane (blue) and out-of-plane (red) 10 nm-thick layers on Au substrate calculated by the electrostatic method. **c** Same as in **b**, but calculated with the electrodynamic method. **d** Same as in **a**, but calculated by the Wave Optics COMSOL Module.

We calculate the near-field spectra presented in Figs. 2 and 3 of the main text using the AC/DC Module of COMSOL Multiphysics[1]. While this electrostatic module does not account for electrodynamic retardation, far-field reflection or propagation effects, it still provides quantitatively accurate results for the near-field spectra. To validate the accuracy of the electrostatic (QE) calculations, we compare them with the standard full-electrodynamical (FED) calculations performed using the Wave Optics COMSOL module[2]. We perform calculations with the two methods for a thin anisotropic layer of 10 nm on a gold substrate (see Fig. S1). In the electrostatic calculations (Fig. S1a,b), the tip is illuminated by a constant vertical electric field $\vec{E}_{\mathrm{inc}} = E_{\mathrm{inc}}\hat{z}$, with magnitude $E_{\mathrm{inc}} = 1$ V/m. For the electrodynamic calculations (Fig. S1c,d), the illumination is a grazing-incident p-polarized plane wave with 1 V/m amplitude and the far-field reflection is neglected.

A comparison of the calculated near-field spectra with both methods (Fig. S1b and Fig. S1c), shows good quantitative agreement in both amplitude and phase for the isotropic layer (black), the layer with in-plane vibrations (blue), and the layer with out-of-plane vibrations (red). We only find a slight reduction in the amplitude of the electrodynamical near-field spectra, explained by radiative losses which reduce the induced dipole moment in the tip. This radiative effect is also observed in the near field distributions around the tip (compare Fig. S1a and Fig. S1d). In the

electrodynamic distribution (Fig. S1d), we observe more spatially spread near fields, with stronger fields around the apex and upper part of the tip. This corresponds to a higher radiation of fields from the tip in the electrodynamic regime.

Section 2: Comparison of near-field spectra of thin layers on different substrates

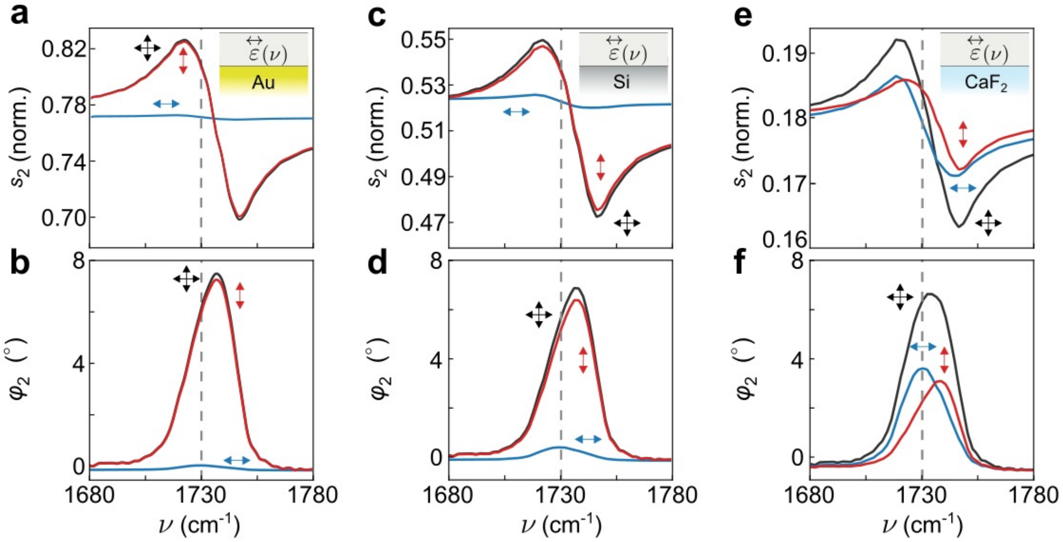

Figure S2. Comparison of the infrared near-field spectra of anisotropic thin layers on different reflecting substrates. **a** Calculated near-field amplitude $s_2(\nu)$ and **b** phase $\varphi_2(\nu)$ spectra of a 5 nm-thin layer with $\overleftrightarrow{\varepsilon}_{\text{iso}}(\nu)$ (black), $\overleftrightarrow{\varepsilon}_{\text{in}}(\nu)$ (blue) and $\overleftrightarrow{\varepsilon}_{\text{out}}(\nu)$ (red) dielectric tensors on an Au substrate. The vertical dashed line indicates the molecular vibration frequency around $\nu = 1730$ cm^{-1}. Black, blue and red arrows represent the orientation of the vibration. Inset in panel **a** show the molecular layer (in light grey) on top of the Au substrate (in yellow). **c, d** Same as in **a, b**, but for 5 nm-thin anisotropic layers on a Si substrate. Inset in panel **c** shows the molecular layer (in light grey) on top of the Si substrate (in dark grey). **e, f** Same as in **a, b**, but for 5 nm-thin anisotropic layers on a CaF_2 substrate. Inset in panel **e** shows the molecular layer (in light grey) on top of the CaF_2 substrate (in blue).

Section 3: Electric field lines for thin anisotropic layers

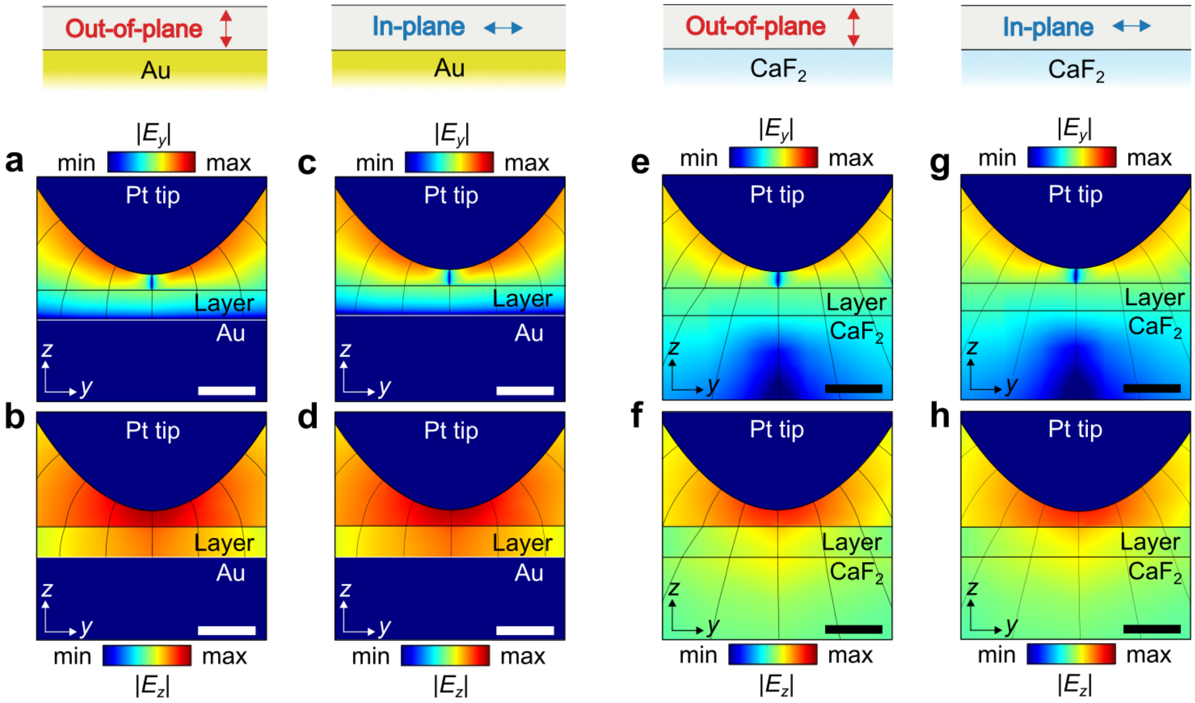

Figure S3. Near-field distribution within anisotropic thin layers on highly and weakly reflecting substrates. **a** Color plot of the amplitude of the in-plane near-field component $|E_y|$, and **b** of the out-of-plane near-field component $|E_z|$. The field plots are calculated around the Pt tip apex above a 10 nm-thin layer with out-of-plane molecular vibrations at $\nu = 1730$ cm^{-1} on an Au substrate. Black lines in **a-b** represent the electric field lines. **c, d** Same as in **a, b**, but for a 10 nm-thin layer with in-plane vibrations. **e-h** Same as in **a-d**, but for layers on a CaF_2 substrate. Scale bar is 20 nm. Color bar range is from min = 0 V/m to max = 50 V/m.

Section 4: Numerical calculations where the tip is modeled as an electric-point dipole

A. Details of the model

To corroborate the peak shifts observed in the near-field spectra presented in Fig. 5 of the main text, we model the metallic tip as an electric-point dipole located above the multilayer system (see Fig. S4). We assume that the vertical dipole $p_0(\nu)$ induced in the tip by the incident field $\vec{E}_{\text{inc}} = E_{\text{inc}}\hat{z}$ (with $E_{\text{inc}} = 1$ V/m) can be described by an electric-point dipole with isotropic polarizability

$$\alpha_{\text{tip}}(\nu) = 4\pi R^3 \varepsilon_0 \frac{\varepsilon_{\text{tip}}(\nu) - 1}{\varepsilon_{\text{tip}}(\nu) + 2}, \quad \text{(S1)}$$

where ν is wavenumber, ε_0 is the permittivity of free space, and $\varepsilon_{\mathrm{tip}}(\nu)$ denotes the dielectric permittivity of the platinum tip with an apex radius R. The induced dipole moment can be determined as:

$$p_0(\nu) = \alpha_{\mathrm{tip}}(\nu) E_{\mathrm{inc}} \, . \qquad \text{(S2)}$$

As shown in Fig. S4, the point dipole is located in air (labeled as region 1) at a distance $z_0 = R + h$ above a semi-infinite slab that models the molecular layer (labeled as region 2). This slab has a thickness d and is characterized by a uniaxial dielectric tensor $\overleftrightarrow{\varepsilon}_2 = \mathrm{diag}[\varepsilon_{xx}, \varepsilon_{xx}, \varepsilon_{zz}]$, which corresponds to the artificial samples with dielectric tensors $\overleftrightarrow{\varepsilon}_{\mathrm{iso}}(\nu)$, $\overleftrightarrow{\varepsilon}_{\mathrm{in}}(\nu)$ and $\overleftrightarrow{\varepsilon}_{\mathrm{out}}(\nu)$ depicted in Fig. 1d of the main text. The anisotropic layer is placed on a semi-infinite substrate layer (labeled as region 3), characterized by a dielectric function ε_3,which represents either gold (Au) or calcium fluoride (CaF_2).

The illuminated point dipole interacts with the multilayer system via its fields $\vec{E}_{\mathrm{s}}(\vec{r}, \nu)$ reflected at the surface of the multilayer sample. Together with the incident field $\vec{E}_{\mathrm{inc}} = E_{\mathrm{inc}}\hat{z}$, the reflected fields produce predominantly, in the point dipole, a net vertical dipole moment

$$p_z(z_0, \nu) = \alpha_{\mathrm{tip}}(\nu)\left[E_{\mathrm{inc}} + E_{\mathrm{s},z}(\vec{r}_0, \nu)\right] , \qquad \text{(S3)}$$

where $E_{\mathrm{s},z}(\vec{r}, \nu)$ represents the z-component of the reflected field evaluated at the position of the point dipole $\vec{r}_0 = (0,0,z_0)$. Thus, the dipole moment p_z depends on the height z_0 of the point dipole relative to the surface of the multilayer system. The reflected fields at the position of the point dipole can be derived from the z-component $G_{zz}(\vec{r}_0, \vec{r}_0, \nu)$ of the Green's tensor of the multilayer system according to the following relationship[3]:

$$E_{\mathrm{s},z}\,(\vec{r}_0, \nu) = \omega^2 \mu_0 G_{zz}(\vec{r}_0, \vec{r}_0, \nu) p_z(z_0, \nu) \, . \qquad \text{(S4)}$$

Here, $\omega = 2\pi c \nu$ is the angular frequency, c is the speed of light in vacuum, μ_0 is the permeability of free space and we refer to $G_{zz}(\vec{r}_0, \vec{r}_0, \nu)$ as the self-interaction Green's function.

By substituting Eq. (S4) into Eq. (S3), we can write the net vertical dipole moment p_z as:

$$p_z(z_0, \nu) = \alpha_{\mathrm{eff}}(z_0, \nu) E_0 \, , \qquad \text{(S5)}$$

where the effective polarizability $\alpha_{\mathrm{eff}}(z_0, \nu)$ is defined by

$$\alpha_{\mathrm{eff}}(z_0, \nu) = \frac{\alpha_{\mathrm{tip}}(\nu)}{1 - \omega^2 \mu_0 \alpha_{\mathrm{tip}}(\nu) G_{zz}(\vec{r}_0, \vec{r}_0, \nu)} \, . \qquad \text{(S6)}$$

In the framework of the point dipole model, we interpret the tip-scattered field as the radiation emitted by the point dipole, which is directly proportional to $p_z(z_0, \nu)$ or $\alpha_{\mathrm{eff}}(z_0, \nu)$, as indicated by Eq. (S5). To compute $\alpha_{\mathrm{eff}}(z_0, \nu)$, we first derive an analytical expression for $\vec{E}_{\mathrm{s}}(\vec{r}, \nu)$ and subsequently determine $G_{zz}(\vec{r}_0, \vec{r}_0, \nu)$ using Eq. (S4). The following section outlines the details of this derivation.

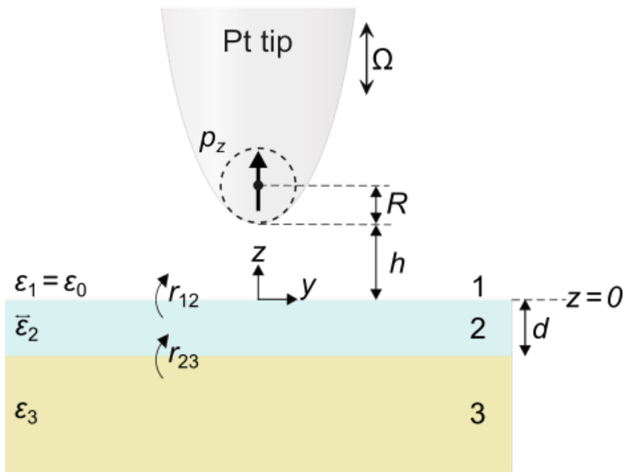

Figure S4. Schematics of the point dipole model. The point dipole, with a vertical dipole moment p_z, is located in air at $\vec{r}_0 = (0,0,z_0 = h + R)$, where R is the tip-apex radius and h is the height between the tip and the molecular layer surface. The dipole oscillates at a frequency Ω above the molecular layer. Regions 1, 2 and 3 refer to air, the molecular layer with thickness d, and the substrate, respectively. Reflection coefficients r_{12} and r_{23} are labelled in the scheme.

B. Calculation of the reflected field and the self-interaction Green's function

To derive an analytical expression of the reflected field $\vec{E}_s(\vec{r},\nu)$, we solve the following inhomogeneous Helmholtz equation, derived from Maxwell's equations[4]:

$$\nabla^2\vec{E}(\vec{r},\nu) + k_0^2\overleftrightarrow{\varepsilon}\cdot\vec{E}(\vec{r},\nu) = -i\omega\mu_0\vec{J}(\vec{r},\nu) + \nabla\left[\nabla\cdot\vec{E}(\vec{r},\nu)\right]. \qquad \text{(S7)}$$

This equation determines the dynamics of the total electric field $\vec{E}(\vec{r},\nu)$ which is the sum of the field produced by the point dipole plus the reflected field. In Eq. (S7), the current density $\vec{J}(\vec{r},\nu) = -i\omega p_z\delta(\vec{r}-\vec{r}_0)\hat{z}$ corresponds to a point dipole located at position $\vec{r}_0 = (0,0,z_0)$, which carries a dipole moment equal to $\vec{p} = p_z\hat{z}$ (see Fig. S4). δ denotes the Dirac delta function.

Owing to the cylindrical symmetry of the system, the total electric field can be decomposed into radial (ρ) and vertical (z) components as $\vec{E}(\rho,z,\nu) = E_\rho(\rho,z,\nu)\hat{\rho} + E_z(\rho,z,\nu)\hat{z}$, with $\hat{\rho} = (\cos\phi\,,\sin\phi\,,0)$ the unitary vector along the radial direction and ϕ the azimuthal angle of the position vector $\vec{r} = (x,y,z)$. In this cylindrical coordinate system, the uniaxial dielectric tensor remains diagonal, retaining its form ($\overleftrightarrow{\varepsilon}_2 = \mathrm{diag}[\varepsilon_{\rho\rho}{=}\varepsilon_{xx}\,, \varepsilon_{\phi\phi} = \varepsilon_{xx}\,, \varepsilon_{zz}]$). Taking advantage of this symmetry, using Gauss's law and performing a spatial-to-momentum Fourier transform to Eq. (S7) with respect to the radial (ρ) and azimuthal (ϕ) cylindrical variables, one can derive the following differential equation for the total electric field:

$$\left(-q^2 + \frac{\partial^2}{\partial z^2}\right)\vec{E}(q,z,\nu) + k_0^2\overleftrightarrow{\varepsilon}\cdot\vec{E}(q,z,\nu) = -i\omega\mu_0 J_z(q,z,\nu)\hat{z} + \frac{1}{i\omega\varepsilon_0}\left(iq\hat{k}_\rho + \hat{z}\frac{\partial}{\partial z}\right)\frac{\partial J_z(q,z,\nu)}{\partial z}. \qquad \text{(S8)}$$

Here $\vec{E}(q,z,\nu) = E_\rho(q,z,\nu)\hat{k}_\rho + E_z(q,z,\nu)\hat{z} = \mathcal{F}[\vec{E}(\rho,z,\nu)]$ and $\mathcal{F}$ denotes the spatial-to-momentum Fourier transform. The wavevector of the field is $\vec{k} = q\hat{k}_\rho + k_z\hat{z}$, with $\hat{k}_\rho = (\cos\phi_k, \sin\phi_k, 0)$ and ϕ_k denotes the azimuthal angle of the wavevector $\vec{k} = (k_x, k_y, k_z)$.

Solving Eq. (S8) separately in the three media, yields the following components of the total electromagnetic field in each region:

$$E_{1\rho}(q,z,\nu) = E_{1\rho}^{+}e^{-\kappa_1 z} - \frac{i}{2}\frac{p_z}{\varepsilon_0} q \,\mathrm{sgn}(z - z_0)\, e^{-\kappa_1|z-z_0|}\,, \tag{S9a}$$

$$E_{1z}(q,z,\nu) = E_{1z}^{+}e^{-\kappa_1 z} + \frac{1}{2}\frac{p_z}{\varepsilon_0}\frac{q^2}{\kappa_1} e^{-\kappa_1|z-z_0|} - \frac{p_z}{\varepsilon_0}\delta(z - z_0)\,, \tag{S9b}$$

$$B_{1\phi}(q,z,\nu) = \frac{1}{\omega}\left(i\kappa_1 E_{1\rho}^{+} - qE_{1z}^{+}\right)e^{-\kappa_1 z} + \frac{1}{2\omega}\frac{p_z}{\varepsilon_0}\left(\kappa_1 q - \frac{q^3}{\kappa_1}\right)e^{-\kappa_1|z-z_0|} + \frac{q}{\omega}\frac{p_z}{\varepsilon_0}\delta(z - z_0) \tag{S9c}$$

$$E_{2\rho}(q,z,\nu) = E_{2\rho}^{-}e^{-i\kappa_o z} + E_{2\rho}^{+}e^{i\kappa_o z} + \frac{q\kappa_e}{k_2^2 - \kappa_e^2}\left(E_{2z}^{-}e^{-i\kappa_e z} - E_{2z}^{+}e^{i\kappa_e z}\right), \tag{S9d}$$

$$E_{2z}(q,z,\nu) = E_{2z}^{-}e^{-i\kappa_e z} + E_{2z}^{+}e^{i\kappa_e z}\,, \tag{S9e}$$

$$B_{2\phi}(q,z,\nu) = \frac{\kappa_o}{\omega}\left(E_{2\rho}^{+}e^{i\kappa_o z} - E_{2\rho}^{-}e^{-i\kappa_o z}\right) - \frac{q}{\omega}\left(\frac{k_2^2}{k_2^2 - \kappa_e^2}\right)\left(E_{2z}^{-}e^{-i\kappa_e z} + E_{2z}^{+}e^{i\kappa_e z}\right), \tag{S9f}$$

$$E_{3\rho}(q,z,\nu) = E_{3\rho}^{-}e^{\kappa_3 z}\,, \tag{S9g}$$

$$E_{3z}(q,z,\nu) = E_{3z}^{-}e^{\kappa_3 z}\,, \tag{S9h}$$

$$B_{3\phi}(q,z,\nu) = -\frac{1}{\omega}\left(i\kappa_3 E_{3\rho}^{-} + qE_{3z}^{-}\right)e^{\kappa_3 z}\,, \tag{S9i}$$

where sgn stands for the sign function and we assume that the EM waves at each medium have wavevectors $\vec{k}_1 = q\hat{k}_\rho + i\kappa_1\hat{z}$, $\vec{k}_e = q\hat{k}_\rho + \kappa_e\hat{z}$, $\vec{k}_o = q\hat{k}_\rho + \kappa_o\hat{z}$ and $\vec{k}_3 = q\hat{k}_\rho + i\kappa_3\hat{z}$, satisfying the subsequent relationships

$$k_1^2 = k_0^2 = \frac{\omega^2}{c^2}\,, \quad \kappa_1^2 = q^2 - k_0^2\,, \tag{S10a}$$

$$k_2^2 = k_0^2\varepsilon_{xx}\,, \quad \kappa_o^2 = k_2^2 - q^2\,, \quad \kappa_e^2 = k_2^2 - \frac{\varepsilon_{xx}}{\varepsilon_{zz}}q^2\,, \tag{S10b}$$

and

$$k_3^2 = k_0^2\varepsilon_3\,, \quad \kappa_3^2 = q^2 - k_3^2\,. \tag{S10c}$$

The subscripts "o" and "e" stand for ordinary and extraordinary waves, respectively, whereas the superscripts "+" and "-" denote waves propagating upward and downward along the vertical axis. The eight coefficients $E_{1\rho}^{+}$, E_{1z}^{+}, $E_{2\rho}^{-}$, $E_{2\rho}^{+}$, E_{2z}^{-}, E_{2z}^{+}, $E_{3\rho}^{-}$, E_{3z}^{-} can be obtained from the application of the standard boundary conditions, together with Gauss's law, for the electromagnetic fields at the interfaces ($z = 0$ and $z = -d$) between the three media, that is,

$$E_{1\rho}|_{z=0} = E_{2\rho}|_{z=0}\ ,\ E_{1z}|_{z=0} = \varepsilon_{zz}E_{2z}|_{z=0}\ ,\ B_{1\phi}|_{z=0} = B_{2\phi}|_{z=0}\ , \tag{S11a}$$

$$E_{2\rho}|_{z=-d} = E_{3\rho}|_{z=-d}\ ,\ \varepsilon_{zz}E_{2z}|_{z=-d} = \varepsilon_3 E_{3z}|_{z=-d}\ ,\ B_{2\phi}|_{z=-d} = B_{3\phi}|_{z=-d}\ , \tag{S11b}$$

$$\left(iq\varepsilon_{xx}E_{2\rho} + \varepsilon_{zz}\frac{\partial E_{2z}}{\partial z}\right)_{z=0} = 0\ ,\ \left(iq\varepsilon_{xx}E_{2\rho} + \varepsilon_{zz}\frac{\partial E_{2z}}{\partial z}\right)_{z=-d} = 0\ . \tag{S11c}$$

From these conditions one finds a linear system of equations that can be expressed as:

$$\mathbb{M}\vec{x} = \vec{b}, \tag{S12}$$

with

$$\mathbb{M} = \begin{bmatrix} 0 & 1 & 0 & 0 & -\varepsilon_{zz} & -\varepsilon_{zz} & 0 & 0 \\ -1 & 0 & 1 & 1 & \frac{q\,\kappa_{\mathrm{e}}}{k_2^2-\kappa_{\mathrm{e}}^2} & -\frac{q\,\kappa_{\mathrm{e}}}{k_2^2-\kappa_{\mathrm{e}}^2} & 0 & 0 \\ \kappa_1 & iq & -i\kappa_{\mathrm{o}} & i\kappa_{\mathrm{o}} & -i\frac{q\,k_2^2}{k_2^2-\kappa_{\mathrm{e}}^2} & -i\frac{q\,k_2^2}{k_2^2-\kappa_{\mathrm{e}}^2} & 0 & 0 \\ 0 & 0 & 1 & 1 & 0 & 0 & 0 & 0 \\ 0 & 0 & 0 & 0 & \varepsilon_{zz}e^{i\kappa_{\mathrm{e}}d} & \varepsilon_{zz}e^{-i\kappa_{\mathrm{e}}d} & 0 & -\varepsilon_3 e^{-\kappa_3 d} \\ 0 & 0 & e^{i\kappa_{\mathrm{o}}d} & e^{-i\kappa_{\mathrm{o}}d} & \frac{q\kappa_{\mathrm{e}}}{k_2^2-\kappa_{\mathrm{e}}^2}e^{i\kappa_{\mathrm{e}}d} & -\frac{q\kappa_{\mathrm{e}}}{k_2^2-\kappa_{\mathrm{e}}^2}e^{-i\kappa_{\mathrm{e}}d} & -e^{-\kappa_3 d} & 0 \\ 0 & 0 & -i\kappa_{\mathrm{o}}e^{i\kappa_{\mathrm{o}}d} & i\kappa_{\mathrm{o}}e^{-i\kappa_{\mathrm{o}}d} & -i\frac{qk_2^2}{k_2^2-\kappa_{\mathrm{e}}^2}e^{i\kappa_{\mathrm{e}}d} & -i\frac{qk_2^2}{k_2^2-\kappa_{\mathrm{e}}^2}e^{-i\kappa_{\mathrm{e}}d} & -\kappa_3 e^{-\kappa_3 d} & iqe^{-\kappa_3 d} \\ 0 & 0 & e^{i\kappa_{\mathrm{o}}d} & e^{-i\kappa_{\mathrm{o}}d} & 0 & 0 & 0 & 0 \end{bmatrix},$$

and

$$\vec{x} = \begin{bmatrix} E_{1\rho}^+ \\ E_{1z}^+ \\ E_{2\rho}^- \\ E_{2\rho}^+ \\ E_{2z}^- \\ E_{2z}^+ \\ E_{3\rho}^- \\ E_{3z}^- \end{bmatrix}, \qquad \vec{b} = \frac{p_z}{2\varepsilon_0}\begin{bmatrix} -\frac{q^2}{\kappa_1}e^{-\kappa_1 z_0} + 2\delta(z_0) \\ iqe^{-\kappa_1 z_0} \\ -i\frac{qk_0^2}{\kappa_1}e^{-\kappa_1 z_0} + i2q\delta(z_0) \\ 0 \\ 0 \\ 0 \\ 0 \\ 0 \end{bmatrix}.$$

By solving the above linear system of equations, using that $k_{1z} = i\kappa_1$, $k_{3z} = i\kappa_3$ and relations in Eq. (S10), one further finds that the coefficient E_{1z}^+ can be expressed in the following compact form:

$$E_{1z}^+ = \frac{p_z}{2\varepsilon_0}\left[i\frac{q^2}{k_{1z}}e^{ik_{1z}z_0}r_{\mathrm{p}} - 2\delta(z_0)\right], \tag{S13}$$

where

$$r_{\mathrm{p}} \equiv r_{\mathrm{p}}(q,\nu) = \frac{r_{12} + r_{23}e^{i2d\kappa_{\mathrm{e}}}}{1 + r_{12}r_{23}e^{i2d\kappa_{\mathrm{e}}}}, \tag{S14}$$

is the total reflection coefficient (p-polarization) of the multilayer system. Here,

$$r_{12} = \frac{k_2^2 k_{1z} - k_0^2 \kappa_\mathrm{e}}{k_2^2 k_{1z} - k_0^2 \kappa_\mathrm{e}}, \qquad \text{and} \qquad r_{23} = \frac{k_3^2 \kappa_\mathrm{e} - k_2^2 k_{3z}}{k_3^2 \kappa_\mathrm{e} - k_2^2 k_{3z}}, \tag{S15}$$

represent the reflection coefficients at the interfaces between medium 2 to 1 (r_{12}) and medium 3 to 2 (r_{23}), respectively (see Fig. S4). Substituting Eq. (S13) into Eq. (S9b), the expression of $E_{1z}(q, z, \nu)$ is then directly obtained as

$$E_{1z}(q, z, \nu) = i \frac{q^2}{k_{1z}} \frac{p_z}{2\varepsilon_0} \left[e^{ik_{1z}(z+z_0)} r_\mathrm{p} + e^{ik_{1z}|z-z_0|} \right] - \frac{p_z}{2\varepsilon_0} \left[\delta(z_0) e^{ik_{1z}z} + \delta(z - z_0) \right]. \tag{S16}$$

Finally, we obtain the *z*-component of the reflected field by subtracting the field produced by the point dipole from Eq. (S16), yielding:

$$E_{\mathrm{s},z}(q, z, \nu) = i \frac{q^2}{k_{1z}} \frac{p_z}{2\varepsilon_0} e^{ik_{1z}(z+z_0)} r_\mathrm{p}. \tag{S17}$$

Using this last expression, along with Eq. (S4), is straightforward to derive the following expression for the *z*-component of the Green's function in the momentum space

$$G_{zz}(q, z, \vec{r}_0, \nu) = \frac{E_{\mathrm{s},z}(q, z, \nu)}{\omega^2 \mu_0 p_z} = \frac{i}{2k_0^2} \frac{q^2}{k_{1z}} e^{ik_{1z}(z+z_0)} r_\mathrm{p}. \tag{S18}$$

Performing a momentum-to-spatial Fourier transform $G_{zz}(q, z, \vec{r}_0, \nu) \longmapsto G_{zz}(\vec{r}, \vec{r}_0, \nu)$, gives the following expressions for the Green's function in real space

$$G_{zz}(\vec{r}, \vec{r}_0, \nu) = \frac{i}{4\pi k_0^2} \int_0^\infty \frac{q^3}{k_{1z}} e^{i[q\rho + k_{1z}(z+z_0)]} \, r_\mathrm{p} \, \mathrm{d}q, \tag{S19}$$

and the self-interaction Green's function

$$G_{zz}(\vec{r}_0, \vec{r}_0, \nu) = \frac{i}{4\pi k_0^2} \int_0^\infty \frac{q^3}{k_{1z}} e^{i2k_{1z}z_0} \, r_\mathrm{p} \, \mathrm{d}q. \tag{S20}$$

We notice that Eqs. (S19) and (S20) differ from those derived in other works[5] in the reflection coefficient r_p. This coefficient considers now a uniaxial layer, whereas the other works consider isotropic layers.

Section 5: Collection of calculated near-field spectra

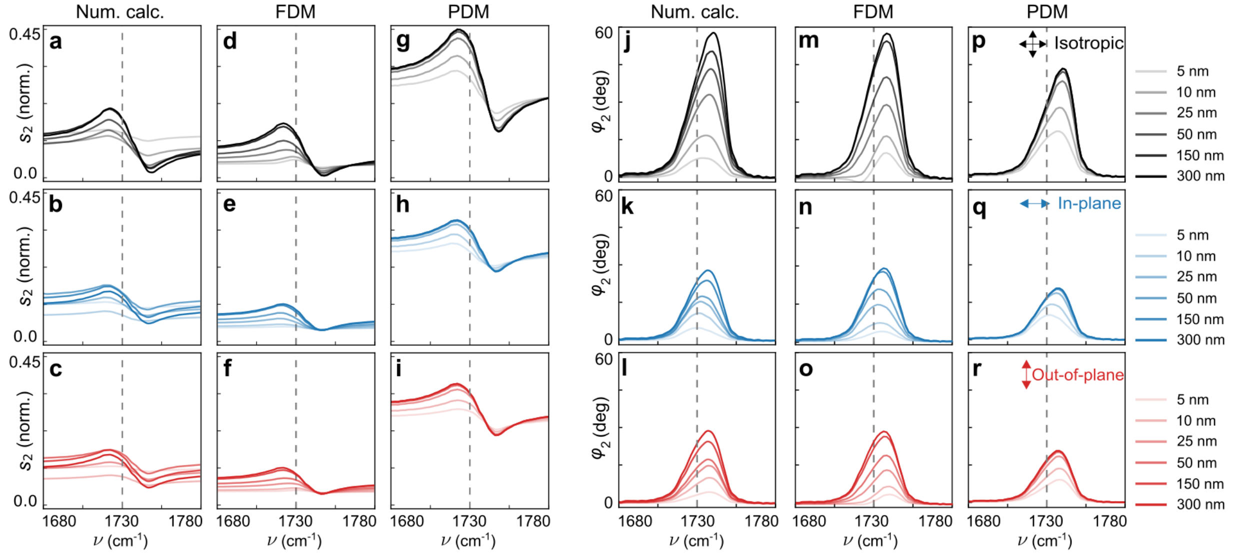

Figure S5. Near-field spectra for layers on CaF_2. a-c Near-field amplitude spectra s_2 for layers (**a** isotropic, **b** in-plane vibrations, **c** out-of-plane vibrations) of different thickness on a CaF_2 substrate calculated with the electrostatic method. **d-f** Same as in **a-c** but calculated with the adapted perturbative FDM. **g-i** Same as in **a-c** but calculated with the $^{eu-}$PDM. **j-r** Near-field phase spectra φ_2 for the same layers in **a-i**. **j-l** Phase spectra calculated with the electrostatic method, **m-o** with the perturbative FDM, **p-r** with the $^{eu-}$PDM.

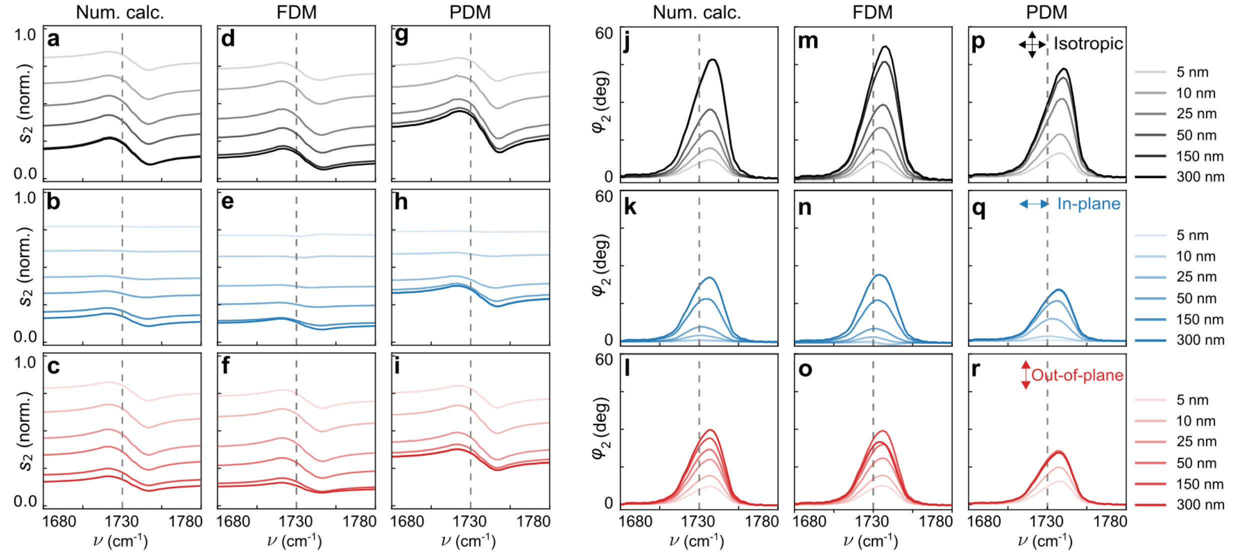

Figure S6. Near-field spectra for layers on Au. a-c Near-field amplitude spectra s_2 for layers (**a** isotropic, **b** in-plane vibrations, **c** out-of-plane vibrations) of different thickness on a gold substrate calculated with the electrostatic method. **d-f** Same as in **a-c** but calculated with the adapted perturbative FDM. **g-i** Same as in **a-c** but calculated with the $^{eu-}$PDM. **j-r** Near-field phase spectra φ_2 for the same layers in **a-i**. **j-l** Phase spectra calculated with the electrostatic method, **m-o** with the perturbative FDM, **p-r** with the $^{eu-}$PDM.

Section 6: Comparison of spectroscopic near-field amplitude and phase contrasts

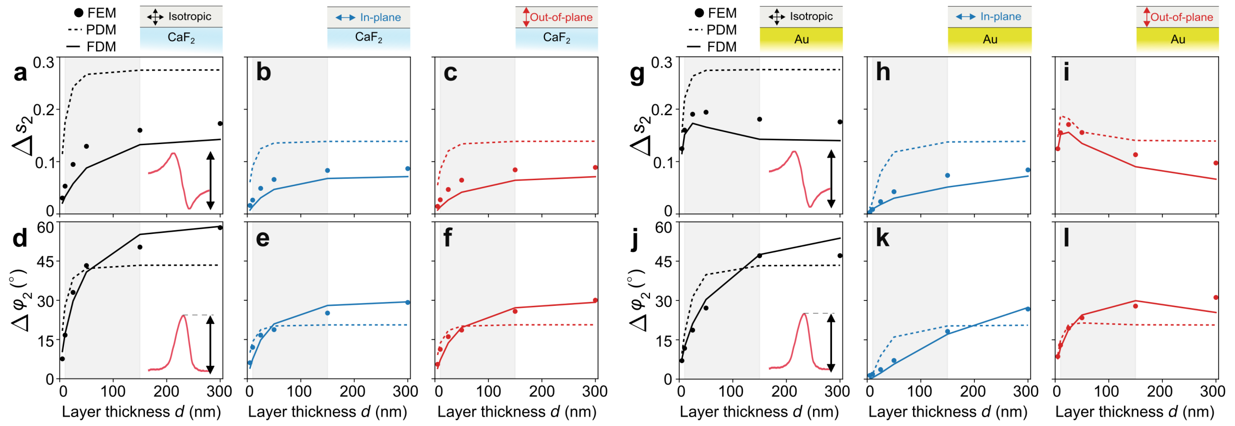

Figure S7. Thickness-dependent spectral near-field contrasts. a-c Spectroscopic amplitude contrast Δs_2 for layers (**a** isotropic, **b** with in-plane vibrations, **c** with out-of-plane vibrations) of different thicknesses on a CaF_2 substrate calculated with the electrostatic method (dots), the ^{eu}PDM (dashed lines), and the perturbative FDM (solid lines). **d-f** Spectroscopic phase contrast $\Delta\varphi_2$ for the same layers and calculation methods as in **a-c**. **g-i** and **j-l** Same as in **a-c** and **d-f** but for layers on a gold substrate, respectively. The shaded grey area corresponds to the layer thickness range where the perturbative FDM is reliable.

Section 7: Phase *vs.* imaginary part of near-field spectra

In this section, we compare φ_2- and $\mathrm{Im}[\sigma_2]$-spectra (Fig. S8), focusing on the spectral contrasts (peak heights) $\Delta\varphi_2$ and $\Delta\mathrm{Im}[\sigma_2]$, and the spectral peak positions $\nu_{\varphi 2}^{\max}$ and $\nu_{\sigma 2}^{\max}$ (Fig. S9).

For layers on CaF_2 substrates, the φ_2- and $\mathrm{Im}[\sigma_2]$-spectra exhibit similar spectral contrasts when normalized to thick layers (Fig. S9a-c), but their peak positions $\nu_{\varphi 2}^{\max}$ and $\nu_{\sigma 2}^{\max}$ differ (Fig. S9g-i). In particular, for thick layers, the peak positions of φ_2-spectra, $\nu_{\varphi 2}^{\max}$, are close to the peaks positions of grazing incidence (GI) FTIR spectra (magenta-dashed line), whereas the peak positions of $\mathrm{Im}[\sigma_2]$-spectra, $\nu_{\sigma 2}^{\max}$, are close to those of transmission (T) FTIR spectra (cyan-dashed line). For thinner layers, the peak positions $\nu_{\varphi 2}^{\max}$ and $\nu_{\sigma 2}^{\max}$ shift and converge. For layers with in-plane vibrations, both peak positions align with that of T-FTIR spectra. For layers with out-of-plane vibrations, they align to that of GI-FTIR spectra. For isotropic layers, they are found slightly above that to T-FTIR spectra.

For thin layers ($d < 100$ nm) on Au substrates, $\mathrm{Im}[\sigma_2]$-spectra exhibits stronger spectral contrast $\Delta\mathrm{Im}[\sigma_2]$ (Fig. S9d-f). In particular, for isotropic layers and layers with out-of-plane vibrations, $\Delta\mathrm{Im}[\sigma_2]$ varies non-monotonically with the layer thickness d, reaching a maximum value at $d \sim 25$ nm. This effect can be explained by substrate-enhanced near-field reflection, which complicates the interpretation of molecular vibrational absorption based on $\mathrm{Im}[\sigma_2]$. Regarding peak positions of φ_2- and $\mathrm{Im}[\sigma_2]$-spectra for layers on Au (Fig. S9j-l), we find a similar behavior

as on CaF_2 substrates for layers with in- and out-of-plane vibrations. For isotropic thin layers, however, both peak positions $\nu_{\varphi 2}^{\max}$ and $\nu_{\sigma 2}^{\max}$ align with that of GI-FTIR spectra rather than to that of T-FTIR spectra.

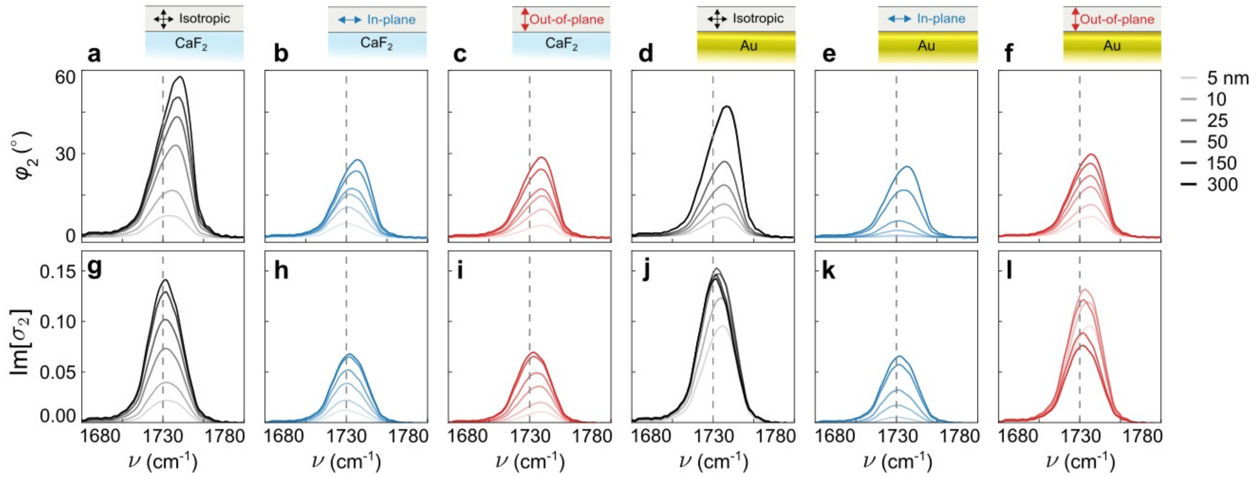

Figure S8. Phase and imaginary part of numerically calculated near-field spectra. a-f Phase of the near-field spectra, φ_2, for layers with isotropic vibrations (**a**, **d**), in-plane vibrations (**b**, **e**), and out-of-plane vibrations (**c**, **f**) on CaF_2 and Au substrates, respectively. **g-l** Imaginary part of the near-field spectra, $\mathrm{Im}[\sigma_2]$, for the same layers as in **a-f**. The vertical dashed lines indicate the molecular vibration at $\nu = 1730$ cm^{-1}.

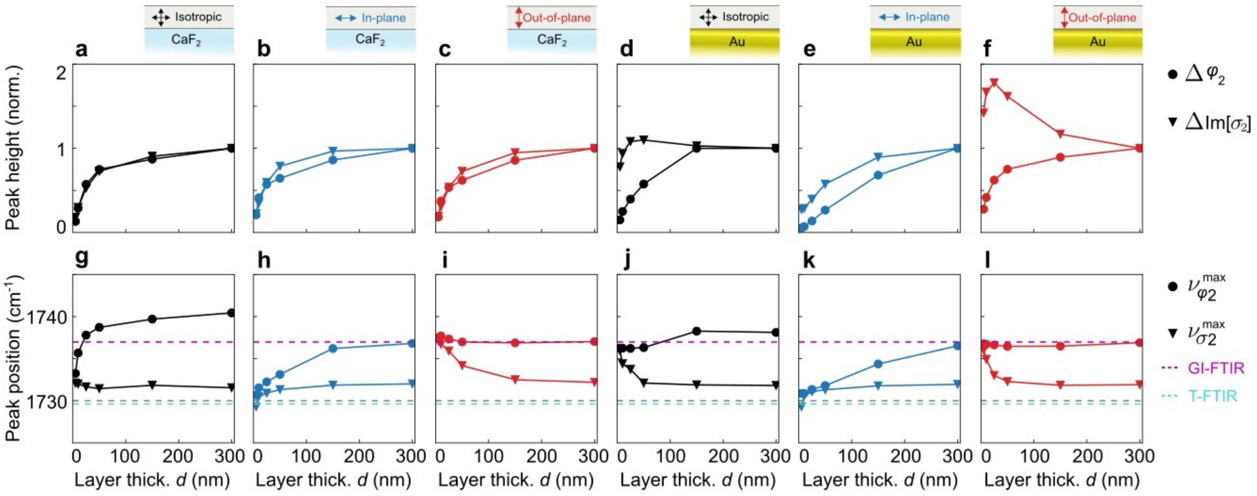

Figure S9. Thickness-dependent height and spectral position of peaks in phase and imaginary part of numerically calculated near-field spectra of thin layers. a-f Spectroscopic contrasts $\Delta\varphi_2$ (dots) and $\Delta\mathrm{Im}[\sigma_2]$ (triangles) for layers with isotropic vibrations (**a**, **d**), in-plane vibrations (**b**, **e**), and out-of-plane vibrations (**c**, **f**) on CaF_2 and Au substrates, respectively. All spectroscopic contrasts are normalized to that of the thickest layer. **g-l** Spectral position of peaks in phase ($\nu_{\varphi 2}^{\max}$, dots) and imaginary part ($\nu_{\sigma 2}^{\max}$, triangles) of near-field spectra of layers with isotropic vibrations (**g**, **j**), in-plane vibrations (**h**, **k**), and out-of-plane vibrations (**i**, **l**) on CaF_2 and Au substrates, respectively. The horizontal, grey-dashed lines indicate the molecular vibration at $\nu = 1730$ cm^{-1}. The horizontal cyan- and magenta-dashed lines indicate the peak position in T-FTIR and GI-FTIR spectra a 10-nm thin layer on a CaF_2 and Au substrate, respectively.

Bibliography of the Supporting Information

(1) COMSOL Multiphysics® v. 5.5. *AC/DC Module User's Guide*; COMSOL AB: Stockholm, Sweden., 2019.

(2) COMSOL Multiphysics® v.5.5. *Wave Optics Module User's Guide*; COMSOL AB: Stockholm, Sweden, 2019.

(3) Novotny, L.; Hecht, B. *Principles of Nano-Optics*, 2nd ed.; Cambridge University Press, 2012.

(4) Chew W. C. *Waves and Fields in Inhomogeneous Media*, 1st ed.; Wiley-IEEE Press, 1995.

(5) Aizpurua, J.; Taubner, T.; García De Abajo, F. J.; Brehm, M.; Hillenbrand, R. Substrate-Enhanced Infrared near-Field Spectroscopy. *Opt. Express* **2008**, *16* (3), 1529–1545.
